# Bioprotective role of *Trichoderma viride* in improving *Abelmoschus esculentus* growth and yield under wastewater irrigation and dual biotic-abiotic stress

**DOI:** 10.3389/fmicb.2026.1920491

**Published:** 2026-09-04

**Authors:** Paras Shah, Yuanhong Wu, Stephan Pollmann, Waqas Raza, Muhammad Shahid Iqbal, Muhammad Abid, Sajid Fiaz, Naitong Yu, Lei Wang, Qurban Ali

**Affiliations:** 1 The Affiliated Sanya Central Hospital of Hainan University, School of Information and Communication Engineering, Hainan University, Haikou, China; 2 Sanya Research Institute of Hainan University, Sanya, China; 3 Centro de Biotecnology Genómica de Plantas, Universidad Politécnica de Madrid (UPM), Instituto Nacional de Investigación y TecnologAgraria y Alimentación (INIA/CSIC), Madrid, Spain; 4 Dingxi Potato Research Institute (General Partnership), Dingxi, Gansu, China; 5 International Potato Center, China Center for Asia Pacific (CIP-CCCAP), Beijing, China; 6 Ministry of Education Key Laboratory for Ecology of Tropical Islands, Key Laboratory of Tropical Animal and Plant Ecology of Hainan Province, College of Life Sciences, Hainan Normal University, Haikou, China; 7 A.G. Lab of Aerobiology and Plant Pathology, Department of Botany, Federal Urdu University of Arts, Science and Technology, Karachi, Pakistan; 8 Institute of Molecular Biology and Biotechnology, The University of Lahore, Lahore, Pakistan; 9 Life Sciences Research Center, Western Caspian University, Baku, Azerbaijan; 10 Institute of Tropical Bioscience and Biotechnology, Chinese Academy of Tropical Agricultural Sciences, Haikou, Hainan, China; 11 Department of Biology, College of Science, United Arab Emirates University, Al-Ain, Abu Dhabi, United Arab Emirates

**Keywords:** antioxidant enzymes, bioprotectant fungus, dual stress, heavy metals, plant growth, plant pathogens, plant yield, wastewater

## Abstract

Irrigation water quality markedly shapes plant growth and physiological functioning, particularly under integrated biotic and abiotic stresses. This study evaluated the influence of irrigation water types, tap water (TW), domestic wastewater (DWW), Lyari wastewater (LWW), and Malir wastewater (MWW), interacting with wastewater-isolated bioprotectant *Trichoderma viride* on *Abelmoschus esculentus* infected with soil-borne pathogens *Fusarium oxysporum* and *Rhizoctonia solani*. Morphological traits, together with ITS amplicon sequencing and BLAST analysis, confirmed *T. viride* (PZ212855). Plants treated with LWW and *T. viride* showed pronounced enhancements in agronomic and physiological traits, i.e., enhanced plant height (101.25 ± 2.87 cm), fresh biomass (24.04 ± 0.86 g), dry biomass (8.45 ± 0.32 g), leaf number (20.25 ± 0.75), fruit fresh biomass (14.33 ± 0.55 g), chlorophyll a (2.12 ± 0.0 4 mg/g F.wt), chlorophyll b (1.30 ± 0.02 mg/g F.wt), total chlorophyll (3.42 ± 0.02 mg/g F.wt), carotenoids (0.75 ± 0.02 mg/g F.wt), and total soluble proteins (1.66 ± 0.02 mg/g F.wt). These increases corresponded with the greater nutrient content of LWW and DWW, which met FAO irrigation standards. DWW upgraded plant functioning, but its slightly higher arsenic concentration required mitigation using *T. viride* in the rhizosphere. MWW, exhibiting greater physicochemical loads and higher arsenic, generated oxidative stress, increased H_2_O_2_ (2.74 ± 0.01 nm/g F.wt) and MDA (0.97 ± 0.03 nm/g F.wt), and reduced growth. *T. viride* partially mitigated these effects by regulating antioxidant enzyme activity. Overall, integrating nutrient-rich wastewater with *T. viride* improved plant growth, yield, and stress resilience under challenging conditions.

## 1 Introduction

Agriculture represents a major consumer of global water resources, with demand escalating due to extreme climatic events that exacerbate resource scarcity, thereby augmenting the vulnerability of food security for the expanding global population (Wu et al., 2024). Consequently, crop production has emerged as a primary concern for agroindustry, as persistent water scarcity continues to adversely impact food production. Researchers are thus actively engaged in developing innovative strategies to ensure that food always remains accessible to all populations, sufficient to support an active and healthy life (Al Aziz et al., 2025; Saadi et al., 2025). According to the World Water Development Report (World Water Development Report (WWDR), 2018), approximately 5 billion individuals will experience water shortages by 2050, with energy and food demand projected to increase by approximately 50% and 40%, respectively (Bwambale et al., 2022). The sustainability of agricultural systems will be compromised if current production patterns persist into the future (Qin and Horvath, 2022). Consequently, the global population faces significant challenges in establishing sustainable food security and transitioning toward achieving the Sustainable Development Goal (SDG) of zero hunger, amidst varying pressures on food production (Erokhin et al., 2022). To sustain food security and meet the demands of a large population, it is imperative to increase crop yields while utilizing 6% of global water resources, suggesting that future environmental and resource challenges will intensify (Huang and Yang, 2017).

In response to global water scarcity, there has been a renewed focus on recycling wastewater for agricultural irrigation. Consequently, concerns regarding water reuse have been revitalized over the past few decades. In 2017, the World Water Development issued an editorial titled “Wastewater: The Untapped Resource,” which documented wastewater as a resource and initiated a transformative shift in water reuse practices (WWAP-UNESCO, 2017). Wastewater, which contains nutrients, has emerged as a sustainable resource to address rising global water demand, thereby supporting sustainable development goals and the global economy (Ricart and Rico, 2019). Its reuse ensures reliable irrigation and enhances crop yields, benefiting farmers on both small and large scales. Previous studies have indicated that recycling wastewater for agricultural irrigation enhances crop output due to its fertilizing properties, which also increases farmers' income by reducing the need for fertilizers (Malki et al., 2017). According to recent studies, wastewater irrigation can substantially lessen fertilizer requirements, with reported reductions of approximately 90% in alfalfa cultivation (Lauriault et al., 2022; Werheni et al., 2025) and around 40–50% in cereal crop production, i.e., wheat, maize, and barley (Saleh et al., 2025). Similarly, other studies have reported savings of approximately €280/ha for tomato production using wastewater (Vergine et al., 2017). Consequently, farmers can achieve significant savings on fertilizer costs, as macronutrients (*N, P*, and K) and micronutrients are readily available in wastewater. However, this issue can be effectively addressed by judiciously utilizing wastewater and ensuring adherence to proper standards (Shah et al., 2022).

Conversely, industrial wastewater is characterized by the presence of various toxic pollutants, including heavy metals (Abbasi and Kamalan, 2018; Shah et al., 2022). The use of such wastewater for crop irrigation results in reduced plant biomass and productivity (Bañón et al., 2011; Lyu et al., 2016). This is attributed to the fact that heavy metals in irrigation water impede the uptake of essential nutrients within the plant and disrupt their translocation from roots to shoots, thereby interfering with metabolic functions and ultimately affecting plant growth (Zhang et al., 2026). Nevertheless, due to water scarcity, farmers are also compelled to use industrial wastewater for agricultural purposes. Consequently, wastewater used for agricultural irrigation must meet minimum standards for specific pollutants, and/or innovative methods must be employed to prevent crop contamination from wastewater use.

To address this issue, a variety of traditional methods have been utilized to remove heavy metals from wastewater, including filtration, chemical precipitation, ion exchange, evaporation, reverse osmosis, membrane technology, carbon adsorption, electrowinning, coagulation, chelation, redox, and electrochemical applications (Cai et al., 2016; da Rocha Ferreira et al., 2019; Khan et al., 2019). However, researchers have identified limitations, cost inefficiencies, and complexities associated with these techniques. Additionally, they contribute to secondary pollution and alter the physical and chemical properties of the environment. According to some studies, many of these technologies are not only costly for large-scale operations, particularly in developing countries, but also pose challenges for continuous monitoring and regulation due to their partial and unpredictable removal of heavy metals (da Rocha Ferreira et al., 2019). They lack precision in their metal-binding capabilities (Ayangbenro and Babalola, 2017). Moreover, they inadvertently eliminate valuable microbial biota and other fauna strains (Li et al., 2025). Consequently, to overcome these limitations, significant attention has been directed by scholars and scientists toward metal-microbe interactions to identify suitable strategies for addressing and mitigating heavy metals in water bodies, soils, and effluents (Li et al., 2025; Shah et al., 2022). This approach is characterized by a short operation time, environmental friendliness, and cost-effectiveness (da Rocha Ferreira et al., 2019; Basheer et al., 2022; Shah et al., 2022).

Microorganisms such as fungi possess the biosorption capacity, enabling them to detoxify and remove heavy metals from their environment (Li et al., 2025; Shao et al., 2025). This process involves both intracellular accumulation within their living cells and extracellular binding through both living and dead biomass (Shao et al., 2025). Researchers have noted that fungal cell walls contain a higher proportion of constituents conducive to metal binding compared to other biosorption agents, thereby enhancing their metal-binding capabilities (Chen et al., 2025a). Numerous studies have explored the removal of heavy metals from wastewater using fungi, which act as metal sinks through biosorption to biomass or to the hyphae (Chen et al., 2025a; Senol et al., 2025).

Many of these fungi also exhibit antagonistic properties against plant pathogens, secreting antibiotics and enzymes such as chitinases, glucanases, and proteases (Shah et al., 2020, 2024; Gao et al., 2022; Huang et al., 2022). Additionally, they have been identified as plant growth promoters, with species such as *Trichoderma* spp. being well-known bioprotectants of several phytopathogenic fungi (Marra et al., 2021; Chohan et al., 2019). This fungus secretes antimicrobial compounds (Vicente et al., 2022) that affect the mycelium of plant-pathogenic fungi, including *Rhizoctonia solani* and *Fusarium oxysporum* f. sp. *lycopersici* (Natsiopoulos et al., 2022). As a bioprotectant, this fungus inhabits and colonizes internal plant tissues without causing chronic symptoms in the host and provides various direct or indirect benefits. These benefits include the acquisition of nutrients, the production of secondary metabolites, antibiotics, cell wall-degrading enzymes, phytohormones, and siderophores, as well as enhanced resistance responses and the development of defense mechanisms against abiotic and biotic stresses. Bioprotectant *Trichoderma viride* strains have been reported in a wide range of hosts and can thrive under diverse environmental conditions (Rai and Solanki, 2023).

Consequently, the study aims to evaluate the potential application of wastewater and wastewater-isolated bioprotectant fungus to improve plant defense mechanisms, thereby mitigating the detrimental impacts of heavy metal-derived stress and increasing plant tolerance to pathogen exposure. Following this rationale, the central hypothesis of the present study is that integrating nutrient-laden wastewater with *T. viride* improves the growth, yield, and stress resilience of *Abelmoschus esculentus* under combined biotic and abiotic stress conditions. Thus, based on prior studies that reported wastewater irrigation and fungal-mediated tolerance, respectively, the present work determines their synergistic application. This strategy is intended to facilitate sustainable approaches for improving crop performance and yield in wastewater irrigation systems.

## 2 Materials and methods

### 2.1 Study site and collection of wastewater samples

**Figure 1:**
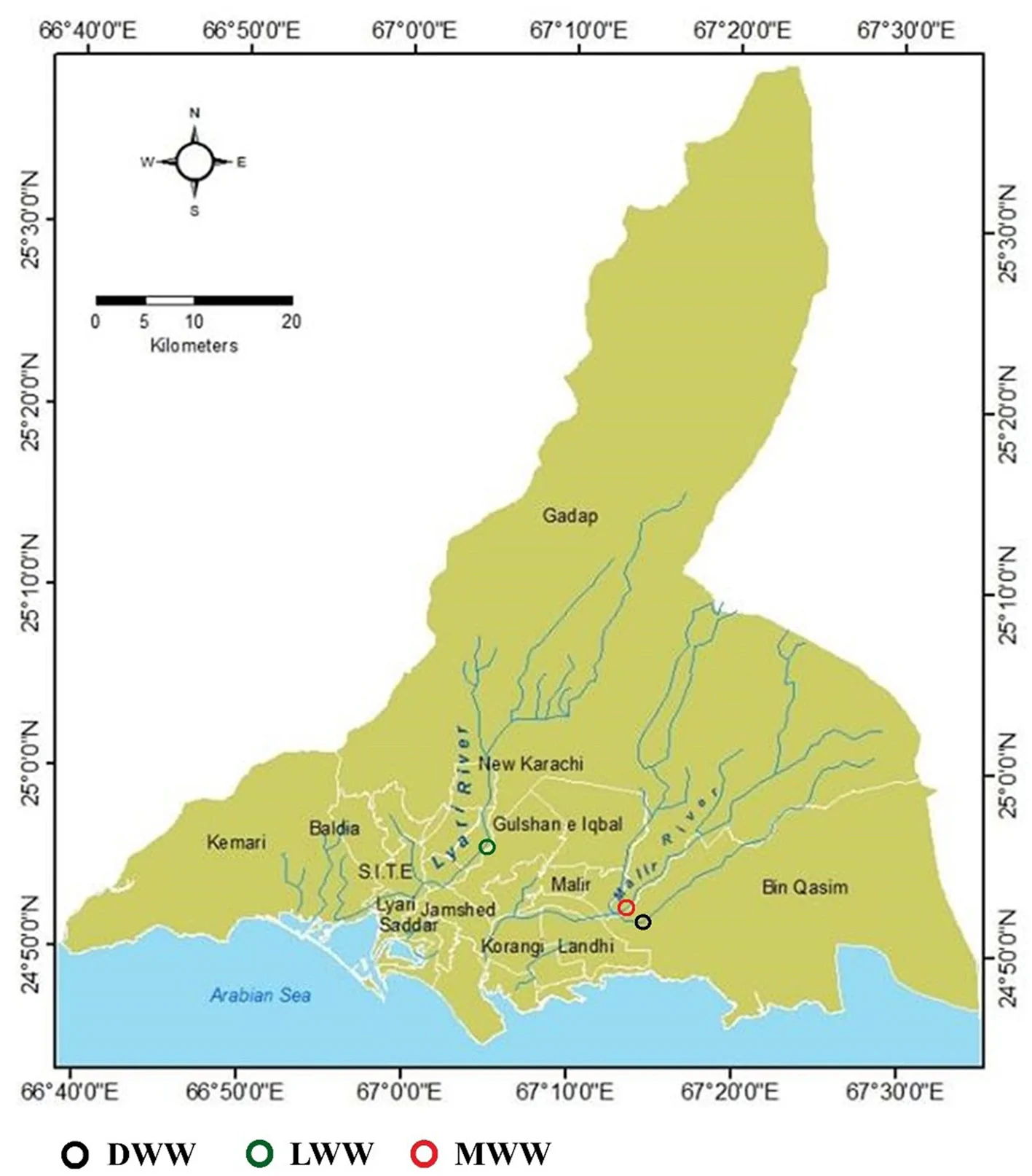
Study area map originated by ArcGIS. Malir and Lyari rivers from origin to end, shown by blue lines.

The Malir and Lyari rivers are the principal watercourses that traverse Karachi, Pakistan. These rivers pre-dominantly depend on rainfall to transport significant volumes of water. However, for most of the year, they are required to convey domestic and industrial wastewater. Notably, wastewater from these rivers is used for irrigation, supporting the cultivation of vegetables, fodder, and ornamental plants in adjacent agricultural fields. In the current study, four distinct types of wastewater were collected for analysis: tap water (TW), domestic wastewater (DWW) originating from households, livestock, and poultry farms, and the wastewaters from the Lyari River (LWW) and Malir River (MWW), as depicted in Figure 1.

Wastewater samples were collected from selected sites using plastic containers with tightly sealed caps. These samples were then transported to the A.G. Laboratory of Plant Pathology and Aerobiology, Department of Botany, Federal Urdu University of Arts, Science and Technology, Karachi, where they were preserved at a temperature of 5–8 °C until required for experimentation.

### 2.2 Water quality analysis

Wastewater samples were subjected to analysis for their physicochemical properties, including pH, electrical conductivity (EC), and total dissolved solids (TDS), utilizing a multi-spectrophotometer model HI9828 (Hanna Instruments, Woonsocket, RI, USA). Additionally, total suspended solids (TSS), dissolved oxygen (DO), biological oxygen demand (BOD), and chemical oxygen demand (COD) were assessed using previously described standard methodologies (APHA, 1998). Elemental analysis was conducted employing the Atomic Absorption Spectrophotometer PE-AAnalyst 700 (PerkinElmer, Springfield, IL, USA). The quantification of elemental concentrations was achieved through the use of appropriate drift blanks and external calibration for precise quantitative analysis.

### 2.3 Isolation and identification of *Trichoderma viride*

*Trichoderma viride* was isolated from wastewater-irrigated soil at an agricultural site (Osman Farm, Quaidabad, Karachi, 24.86286° *N*, 67.22755° E). The *Trichoderma* isolate was first identified based on colony morphology on PDA after incubation at 28 ± 2 °C for 5–7 days, with observations of color, texture, growth pattern, and margins. Microscopic examination evaluated hyphae, conidiophore branching, phialide arrangement, and conidial shape and size, using standard manuals for *T. viride* (Rifai, 1969; Bissett, 1991; Gams and Bissett, 2002). Following morphological identification, the isolate was further grown on *Trichoderma*-Specific Medium (TSM) to obtain the desired biomass. A conidial suspension was made from a 7-day-old culture and standardized to 1 × 10^6^ spores/ml. A standardized suspension (10% v/v) was employed to inoculate 100 ml TSM broth, incubated at 28 ± 2 °C, 150 rpm for 48 h. Later, mycelial biomass was harvested, washed, oven-dried, and utilized for DNA extraction.

Genomic DNA was extracted from a pure culture of *Trichoderma* using the CTAB technique (Sambrook and Russell, 2001). Intended for PCR analysis, the internal transcribed spacer ITS1–5.8S-ITS2 region was amplified using the universal primers ITS1 and ITS4. A single fragment of 550 bp length was amplified and sequenced bi-directionally. Consensus sequences were subjected to BLAST analysis against the NCBI GenBank database, which confirmed the isolate identity as *Trichoderma viride* (Accession: PZ212855). It was further utilized to evaluate its potential for heavy metal tolerance and resistance to pathogenic attacks in plants using a dual culture technique (Shah et al., 2020).

### 2.4 Plant growth experiment

The study employed a completely randomized experimental design to investigate the impact of various wastewaters, i.e., DWW, LWW, and MWW, in comparison to TW irrigation on the growth of *Abelmoschus esculentus* (L.) Moench under field conditions from February to April 2024. Additionally, the experimental framework included an assessment of plant pathogenic diseases under wastewater irrigation, encompassing conditions with no pathogen, *F. oxysporum*, and *R. solani*. The bioprotectant fungal treatment involved either no bioprotectant fungus or *T. viride*.

The experiment used sandy loam soil (composed of 70% sand, 19% silt, and 11% clay) with a moisture-holding capacity of 21% and a total organic matter content of 2.17%. Before the experimental trial, the soil was amended with cow dung manure at a 3:1 soil-to-manure ratio and sterilized through autoclaving. Four sanitized seeds, obtained from the Karachi office of the Department of Seed Certification and Registration, Govt. of Pakistan, were sown in each pot containing 1 kg of sterilized soil, with eight replicates per treatment. At the post-emergence stage, two healthy seedlings of similar size were retained in each pot. The soil was artificially inoculated with a soil-borne phytopathogen suspension (10^8^ CFU) at the first true leaf stage. The isolated phytopathogens and bioprotectant fungal spores were multiplied on PDA. The spores were harvested by flooding sporulating cultures with sterile distilled water, gently scraping the colony surface, and filtering the suspension through sterile muslin cloth to remove mycelial debris. Consequently, a bioprotectant fungal spore suspension (10^8^ CFU) was inoculated 1 week after the pathogenic inoculation. The growing plants were irrigated with the respective wastewater as per the experimental design.

#### 2.4.1 Agronomical traits

After a period of 4 weeks of co-inoculation, plants from each of the replicated pots of treatments were harvested and analyzed for various growth parameters, including the length and weight of both root and shoot, as well as the number of leaves. The remaining four replicate pots per treatment were designated for yield measurements, specifically the number, length, and weight of fruit per plant, which were recorded 75 days post-seed sowing.

#### 2.4.2 Pathogenicity assessment

Additional plants harvested from each pot were utilized for pathogenicity testing and biochemical analysis. The initial manifestation of symptoms, such as chlorosis, necrosis, and wilting, was assessed morphologically to determine the presence of pathogenicity in plants. The assessment was further substantiated by examining the root colonization percentage of pathogens on PDA medium. Root pieces were surface sterilized using 1% sodium hypochlorite (NaOCl) solution for a duration of 2 min. Root pieces were then rinsed three times with sterile distilled water and plated to observe the number of pieces exhibiting pathogen growth. The colonization percentage was then calculated from the observations using the following formula:


$$\begin{array}{l} \text{Colonization \%}=\frac{\text{No. of root cuttings colonized by pathogen}}{\text{Total number of root cuttings}}\\ \;\times 100 \end{array}$$

The percentage of colonization was further quantified using the Root Colonization Index (RCI), which was measured on a 0–5 scale, as established by (Shahzad and Ghaffar 1992).

#### 2.4.3 Biochemical traits

##### 2.4.3.1 Determination of photosynthetic pigments

The chlorophyll content in leaves was analyzed using 80% acetone, following the methodology established by Arnon (1949), while carotenoid analysis adhered to the protocol of Duxbury and Yentsch (1956) using four biological replicates per treatment. Absorbance measurements for chlorophylls were taken at wavelengths of 649 nm and 665 nm, and for carotenoids at 480 nm and 510 nm, utilizing a spectrophotometer (JENWAY 6305, Bibby Scientific, Essex, UK).

##### 2.4.3.2 Total protein contents

Leaf tissues (0.2 g) of each biological quarterly replicates were finely ground in liquid nitrogen and homogenized in 3 ml of potassium phosphate buffer (0.1 M, pH 7) containing 0.1 mm EDTA (Sigma-Aldrich, Burlington, MA, USA) and 1% PVP (Sigma-Aldrich, Burlington, MA, USA). The homogenized samples were subjected to centrifugation at 21,000 × g at 4 °C for 20 min. The resulting supernatant was collected and stored at −20 °C for subsequent analysis of antioxidant enzymes. Using an aliquot of the same homogenate, the total soluble protein content was determined following the Bradford (1976) method.

##### 2.4.3.3 Determination of oxidative damage indicators

Oxidative damage was assessed by measuring lipid peroxidation and hydrogen peroxide (H_2_O_2_) levels in the leaves of each of four biological replicates per treatment. The extract was prepared by homogenizing leaf samples in a 0.1% trichloroacetic acid (TCA) solution, followed by centrifugation at 10,000 × g for 15 min. Lipid peroxidation in the TCA extract was mixed with 1 ml of 0.5% thiobarbituric acid (TBA) assay, which estimates malondialdehyde (MDA) as the final product (Heath and Packer, 1968). The mixture was incubated in a boiling water bath for 30 min, and the reaction was ended by cooling in an ice bath. The absorbance was recorded at 532 nm and adjusted for non-specific turbidity at 600 nm. MDA concentration was calculated using an extinction coefficient of 155 mm^−1^ cm^−1^. For the quantification of H_2_O_2_ content, the TCA extract was mixed with 10 mm potassium phosphate buffer (pH 7.0) and 1 M potassium iodide. The absorbance was recorded at 390 nm by a spectrophotometer (JENWAY 6305, Bibby Scientific, Essex, UK). H_2_O_2_ concentration was then calculated against a standard curve prepared with known H_2_O_2_ concentrations (Sergiev et al., 1997).

##### 2.4.3.4 Quantification of antioxidant enzyme activity

###### 2.4.3.4.1 Superoxide dismutase (SOD)

The activity of superoxide dismutase (EC 1.15.1.1) was assessed following the methodology described by Fridovich (1995) using four biological replicates per treatment. This biocatalytic process is based on the inhibition of the reduction of nitroblue tetrazolium (NBT; Sigma-Aldrich, Burlington, MA, USA) by the superoxide anion (${\text{O}}_{2}^{-}$) generated through riboflavin photoreduction. Solution 1, freshly prepared for each aliquot, contained 50 mm phosphate buffer (pH 7.8), 33 μM NBT, 10 mm methionine (Sigma-Aldrich, Burlington, MA, USA), and 0.6 mm EDTA. In contrast, solution 2 consisted of 20 μl of 0.33 mm riboflavin (Sigma-Aldrich) in 50 mm phosphate buffer (pH 7.8). The reaction mixture, totaling 3 ml (100:4:1), included solution 1, the enzyme extract, and solution 2. The reaction was initiated by exposing the solution to fluorescent light (20 W) for 7 min, while a separate batch was kept in the dark. After the designated time, the reaction products were measured at 560 nm in a spectrophotometer, alongside a non-illuminated reagent blank. An illuminated mixture blank served as a control with a pre-determined absorbance. One unit of SOD activity was defined as the amount of enzyme required to achieve 50% inhibition of NBT reduction.

###### 2.4.3.4.2 Catalase (CAT)

The activity of catalase (E.C. 1.11.1.6) in every four biological replicates per treatment was assessed using the method described by Aebi (1984). The catalytic reaction commences with the reduction of H_2_O_2_, catalyzing its conversion into H_2_O and O_2_. The reaction mixture, with a total volume of 3 ml, comprised 100 mm potassium phosphate buffer (pH 7.0), 30 mm H_2_O_2_, and 0.05 ml of the soluble enzyme fraction. Enzyme activity was quantified by measuring the decrease in absorbance due to the reduction of H_2_O_2_ at 240 nm over a period of 60 s (extinction coefficient = 36 M^−1^ cm^−1^). One unit of catalytic activity at 25 °C was defined as the amount required to reduce 1 μmol of H_2_O_2_ per minute.

###### 2.4.3.4.3 Ascorbate peroxidase (APX)

Ascorbate peroxidase (E.C. 1.11.1.11) activity was determined following the protocol established by Nakano and Asada (1981) using four biological replicates per treatment. The reaction mixture, with a total volume of 3 ml, comprised 50 mm potassium phosphate buffer (pH 7.0), 0.1 mm H_2_O_2_, 0.50 mm ascorbate, and 0.1 ml of the soluble enzyme fraction. The enzymatic activity was evaluated by monitoring the decrease in absorbance due to the oxidation of ascorbate by H_2_O_2_ at 290 nm over a period of 60 s (extinction coefficient = 2.8 M^−1^ cm^−1^).

###### 2.4.3.4.4 Polyphenol oxidase (PPO)

The activity of polyphenol oxidase (E.C. 1.14.18.1) was assessed following the methodology of Sakiroglu et al. (2008), with four biological replicates per treatment. The reaction mixture, with a total volume of 3 ml, comprised 50 mm potassium phosphate buffer (pH 6.5), 50 mm catechol, 2 mm ascorbic acid, and 0.05 ml of the soluble enzyme fraction. The enzyme activity was quantified by monitoring the increase in absorbance at 420 nm at a rate of 0.001 min^−1^, as described by Fenoll et al. (2002).

### 2.5 Statistical analysis

The statistical analysis of plant growth parameters and yield, as well as pathological and enzymatic evaluations, was conducted using two-way analysis of variance (ANOVA) with SPSS (v. 27), in alignment with the research framework. A Tukey *post hoc* test was performed to compare the various treatment modalities.

Correlation analysis and hierarchical clustering were conducted using Origin (2018) to elucidate the interrelationships among the measured growth, yield, and physiological traits of plants in response to different irrigation treatments with the application of a bioprotectant fungus, and to classify these treatments based on their similarity patterns.

## 3 Results

### 3.1 Irrigation water quality indices

The quality of irrigation waters from the various sources described demonstrated marked differences and deviations from the FAO irrigation water quality standards (Table 1). The physicochemical properties, such as pH, electrical conductivity of water (ECw), and total dissolved solids (TDS), in tap water (TW), domestic wastewater (DWW), and Lyari wastewater (LWW) were found to be within the permissible limits set by the FAO standards. Conversely, these parameters exceeded the standard limits in Malir wastewater (MWW). Total suspended solids (TSS) were observed to be higher in DWW (392.33 mg L^−1^), LWW (411.00 mg L^−1^), and MWW (775.33 mg L^−1^) than the permissible range of the FAO, while biochemical oxygen demand (BOD) was only within the limit in TW (14.67 mg L^−1^) and DWW (121.33 mg L^−1^).

**Table 1:** Irrigation-water quality indices of various sources, in comparison with the irrigation water quality standards provided by the National Environmental Quality Standards (NEQS, 2000) and the Food and Agriculture Organization (FAO, Ayers and Westcot, 1994).

Parameters	Units	TW	DWW	LWW	MWW	*F-value*	NEQS	FAO
Physicochemical quality indices								
pH	—	7.600	8.300	8.133	8.967	30.070^***^	6–10	6.5–8.4
		±0.100	±0.058	±0.120	±0.120			
ECw	μs/cm	789	1,596	2,771	3,667	226.839^***^	—	700–3,000
		±8.373	±66.577	±140.951	±63.553			
TDS	mg/L	475.000	1,052.333	1,731.333	2,214.333	82.331^***^	3,500	450–2,000
		±4.583	±45.171	±62.130	±149.481			
TSS	mg/L	58.000	392.333	411.000	775.333	1,260.576^***^	150	350
		±1.732	±9.387	±5.774	±12.170			
DO	mg/L	0.000	0.490	0.673	1.033	354.204^***^	—
	±0.000	±0.012	±0.020	±0.039			
BOD	mg/L	14.667	121.333	364.333	717.667	2,366.271^***^	80	300
		±0.333	±1.453	±12.238	±3.528			
COD	mg/L	155.000	239.000	412.333	1,047.333	414.480^***^	150
	±2.309	±11.136	±5.925	±37.534			
Macronutrients								
K	mg/L	0.098	8.217	4.140	11.500	217.606^***^	—	0–2
	±0.000	±0.577	±0.154	±0.305				
Ca	mg/L	0.847	92.590	52.937	74.880	760.911^***^	—	0–400
		±0.019	±1.516	±1.287	±2.088			
Mg	mg/L	0.230	23.640	9.403	15.073	338.594^***^	—	0–60.75
		±0.017	±0.915	±0.356	±0.422			
Micronutrients								
Fe	mg/L	BDL	BDL	BDL	BDL	—	2.0	5.0
Zn	mg/L	0.037	0.043	0.094	BDL	167.517^***^	5.0	2.0
		±0.003	±0.003	±0.004				
Cu	mg/L	0.001	0.005	0.015	0.003	66.467^***^	1.0	0.2
		±0.000	±0.000	±0.001	±0.000			
Heavy metals								
Pb	mg/L	BDL	BDL	BDL	BDL	—	0.5	5.0
As	mg/L	0.002	0.185	BDL	0.303	4,481.000^***^	1.0	0.1
		±0.000	±0.002	±0.004				
Ni	mg/L	BDL	0.004	BDL	0.021	248.262^***^	1.0	0.2
			±0.000	±0.001				
Cd	mg/L	BDL	0.002	0.002	0.004	56.667^***^	0.1	0.01
			±0.000	±0.000	±0.000			

TW, tap water; DWW, domestic wastewater; LWW, Lyari wastewater; MWW, Malir wastewater; NEQS, national environmental quality standards (water quality standards); FAO, food and agriculture organization (irrigation water quality for agriculture); BDL, below the detection limit; ±, standard error of replicates; Sig. (P), significance level: ^*^P < 0.05; ^**^P < 0.01 and ^***^P < 0.001.

In contrast, macronutrients such as potassium, magnesium, and calcium were more abundant in DWW, LWW, and MWW compared to TW. However, micronutrients like iron, zinc, and copper in all irrigation waters were found to be within the range of the FAO guidelines (Table 1), although higher concentrations of micronutrients were observed in all wastewaters compared to TW. Conversely, heavy metals, including lead, nickel, and cadmium, were also found to be within the permissible limits in all irrigation waters. Nonetheless, higher concentrations of arsenic were detected in DWW (0.185 mg L^−1^) and MWW (0.303 mg L^−1^) than the FAO standards for irrigation water.

**Figure 2:**
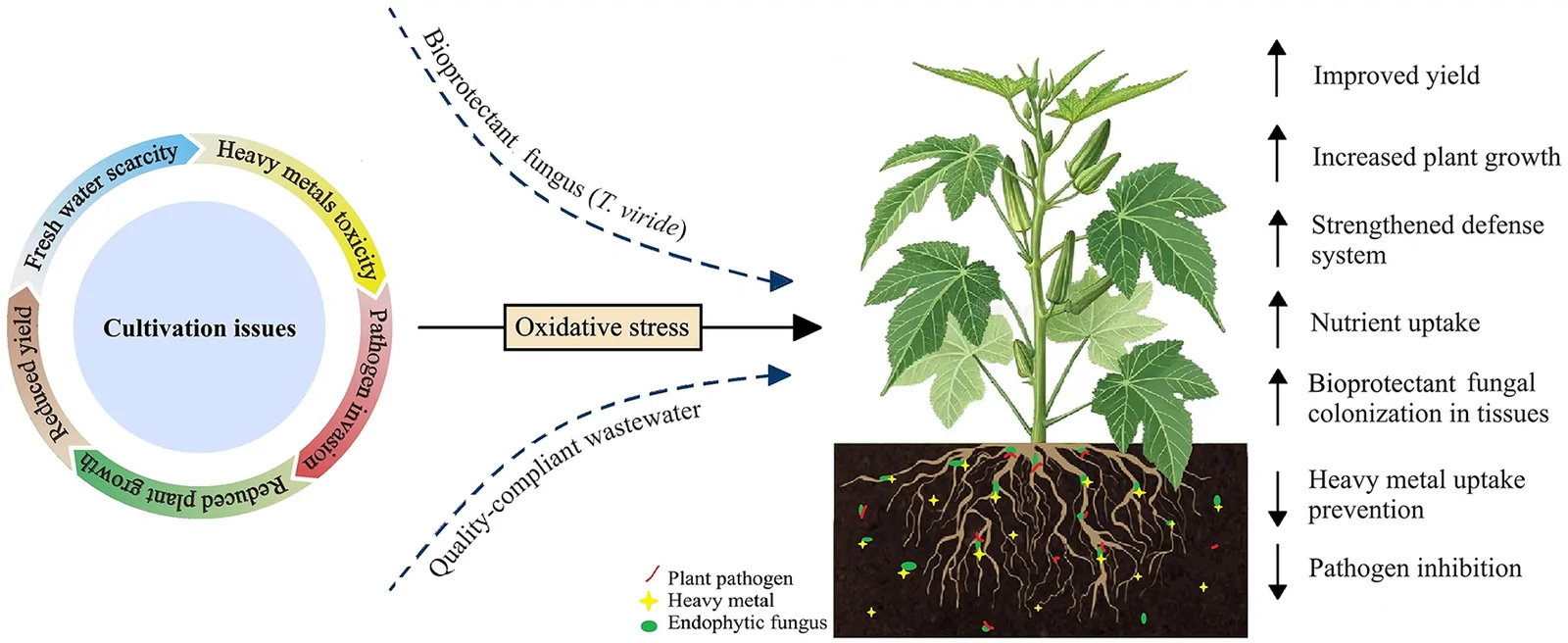
Overview of cultivation-related stress dynamics and the protective role of bioprotectant *Trichoderma viride* in improving plant growth, defense responses, and yield while decreasing pathogen and heavy metal effects.

Overall, the water sources primarily differed in their physicochemical parameters. All essential nutrients were found to be higher in DWW, LWW, and MWW than in TW. While heavy metals were within the standard limits in all water sources, arsenic concentrations were higher in MWW and DWW. These distinctions indicate that DWW and LWW are suitable for crop irrigation under FAO standards guidelines. Whereas MWW falls short of the standards due to its elevated contaminant load. Thus, the suitability of wastewater for irrigation based on its source and quality requires individual evaluation rather than broad generalization (Figure 2).

### 3.2 Morphological and molecular identification of *Trichoderma viride*

The *Trichoderma* isolate was collected from wastewater-irrigated agricultural soil at Osman Farm (Quaidabad, Karachi). The isolate was initially identified based on its rapid growth on PDA, reaching 70–80 mm in diameter after 7 days at 28 ± 2 °C. Colonies were primarily white, later bright green to dark green during conidiation. The colony surface appeared cottony, with distinct concentric rings. Colony margins were smooth, and the reverse side was cream-colored without pigment diffusion. Microscopic investigation showed branched, septate, and hyaline hyphae. Conidiophores were vastly branched, developing pyramidal structures. Flask-shaped phialides were usually organized in divergent whorls and were about 7–10 μm in length. Conidia were globose to subglobose, smooth-walled, and green, with a diameter of 3–4 μm. Revealed morphological characteristics conformed to the well-known descriptions of *T. viride*.

For molecular identification, genomic DNA extracted using the CTAB method was sufficiently pure for PCR amplification. BLAST analysis of the ITS region identified the isolate as *T. viride* (≥99% identity, 100% coverage). The ITS1 amplicon sequence of the isolate was submitted to GenBank under accession number PZ212855.

### 3.3 Agronomical traits

In the performed *in vivo* study, it was observed that DWW and LWW significantly enhanced agronomic traits, including growth parameters and yield, in the test plants. Conversely, MWW adversely affected the growth and yield of *Abelmoschus esculentus* (L.) Moench (Tables 2, 3).

**Table 2:** Growth-related agronomic traits of *Abelmoschus esculentus* (L.) Moench, subjected to various irrigation water qualities and pathogenic stress with the interaction of the bioprotectant fungus *T. viride*.

Parameters	Irrigation	Treatments						
		Control	*F. oxysporum*	*R. solani*	*T. viride*	*F. oxysporum* + *T. viride*	*R. solani* + *T. viride*	
Root length (cm)	TW	18.25	11.25	9.25	28.50	23.00	20.25	b
		±0.75	±0.75	±0.85	±1.04	±1.29	±0.85	
	DWW	23.00	14.25	12.00	33.75	27.25	28.75	c
		±1.58	±1.11	±0.91	±1.38	±1.25	±0.63	
	LWW	25.75	17.25	16.25	38.25	34.25	31.50	d
		±1.11	±1.11	±1.11	±1.49	±1.49	±1.32	
	MWW	12.25	8.00	13.75	18.75	14.50	16.50	a
		±0.63	±0.58	±0.63	±1.11	±0.65	±1.19	
	b	a	a	d	c	c
Shoot length (cm)	TW	34.00	22.50	19.25	49.00	32.25	28.75	a
		±1.58	±1.32	±1.65	±3.49	±1.70	±1.70	
	DWW	45.50	32.00	27.75	57.00	54.25	45.75	b
		±1.32	±1.29	±1.93	±1.83	±1.65	±2.02	
	LWW	51.75	38.00	34.00	63.00	48.50	58.00	c
		±3.40	±1.29	±1.96	±2.94	±1.71	±2.68	
	MWW	23.00	19.25	30.25	33.75	27.75	34.25	a
		±1.96	±1.93	±2.02	±2.39	±1.80	±1.80	
	b	b	b	c	b	b
Root fresh weight (g)	TW	1.93	1.03	0.78	2.40	1.98	1.85	b
		±0.11	±0.11	±0.03	±0.09	±0.08	±0.03	
	DWW	2.53	1.70	1.53	3.53	2.83	2.53	c
		±0.15	±0.09	±0.14	±0.10	±0.03	±0.06	
	LWW	2.93	1.95	1.65	3.93	3.18	2.75	d
		±0.11	±0.10	±0.09	±0.06	±0.14	±0.03	
	MWW	0.93	0.65	0.88	1.68	1.10	1.53	a
		±0.09	±0.06	±0.05	±0.08	±0.09	±0.05	
	b	a	a	d	c	bc
Shoot fresh weight (g)	TW	11.84	8.12	6.12	14.52	10.79	9.18	b
		±0.59	±0.66	±0.41	±0.68	±0.50	±0.73	
	DWW	12.88	10.44	10.05	17.69	14.55	12.98	c
		±0.56	±0.76	±0.45	±0.81	±0.90	±0.58	
	LWW	14.37	11.11	9.79	20.14	16.58	13.14	d
		±0.63	±0.67	±0.57	±0.87	±0.50	±0.71	
	MWW	7.66	4.40	5.82	10.11	6.97	8.47	a
		±0.66	±0.44	±0.24	±0.97	±0.42	±0.53	
	b	a	a	c	b	b
Root dry weight (g)	TW	0.88	0.42	0.33	1.24	0.84	0.72	b
		±0.03	±0.02	±0.02	±0.03	±0.03	±0.05	
	DWW	1.67	0.81	0.78	2.22	1.82	1.78	c
		±0.04	±0.02	±0.02	±0.12	±0.03	±0.05	
	LWW	2.12	0.77	0.74	2.71	2.33	1.98	d
		±0.07	±0.03	±0.02	±0.18	±0.09	±0.10	
	MWW	0.35	0.11	0.25	0.98	0.31	0.45	a
		±0.02	±0.01	±0.02	±0.05	±0.02	±0.03	
	b	a	a	c	b	b
Shoot dry weight (g)	TW	3.11	2.46	1.87	3.80	3.12	2.94	b
		±0.19	±0.23	±0.09	±0.19	±0.17	±0.11	
	DWW	3.89	3.34	2.94	5.10	4.74	4.32	c
		±0.31	±0.28	±0.11	±0.20	±0.19	±0.29	
	LWW	4.20	3.53	3.10	5.72	4.21	4.15	c
		±0.26	±0.24	±0.12	±0.20	±0.14	±0.26	
	MWW	1.74	0.71	1.21	2.91	1.43	1.92	a
		±0.03	±0.06	±0.16	±0.14	±0.10	±0.10	
	b	a	a	c	b	b
Number of leaves per plant	TW	11.25	9.00	7.25	15.75	13.25	10.75	b
		±0.48	±0.71	±1.03	±1.31	±0.75	±0.85	
	DWW	14.75	11.75	11.50	21.00	16.25	13.50	c
		±1.38	±1.38	±1.04	±0.91	±1.31	±1.19	
	LWW	17.25	12.75	10.25	20.25	19.00	12.25	c
		±1.38	±1.03	±0.48	±0.75	±1.08	±0.85	
	MWW	8.25	8.75	7.75	11.00	9.25	9.75	a
		±0.75	±1.44	±0.75	±1.22	±0.48	±0.63	
	cd	ab	a	e	d	bc

TW, tap water; DWW, domestic wastewater; LWW, Lyari wastewater; MWW, Malir wastewater; ±, standard error of replicates; means followed by letters vary significantly based on Two-Way ANOVA; Tukey's HSD test (*p* < 0.05).

**Table 3:** Yield-related agronomic traits of *Abelmoschus esculentus* (L.) Moench, subjected to various irrigation water qualities and pathogenic stress with the interaction of the bioprotectant fungus *T. viride*.

Parameters	Irrigation	Treatments						
Control	*F. oxysporum*	*R. solani*	*T. viride*	*F. oxysporum* + *T. viride*	*R.solani* + *T. viride*
Number of fruits per plant	TW	3.50	1.50	1.25	6.25	3.25	3.00	b
		±0.29	±0.29	±0.25	±0.25	±0.25	±0.00	
	DWW	5.25	3.25	2.50	6.25	3.50	3.75	c
		±0.25	±0.25	±0.29	±0.25	±0.29	±0.25	
	LWW	5.75	3.50	2.50	6.50	3.50	3.50	c
		±0.25	±0.29	±0.29	±0.29	±0.29	±0.29	
	MWW	1.00	1.00	1.25	2.50	1.75	2.75	a
		±0.00	±0.00	±0.25	±0.29	±0.25	±0.25	
	c	a	a	d	b	b
Fruit length (cm)	TW	10.02	6.78	4.65	13.21	9.26	7.74	b
		±0.68	±0.40	±0.42	±0.54	±0.72	±0.60	
	DWW	10.44	8.83	7.53	14.10	11.89	12.78	c
		±0.68	±0.52	±0.27	±0.34	±0.84	±0.74	
	LWW	12.38	9.04	8.14	12.95	10.14	11.14	c
		±0.61	±0.37	±0.49	±0.47	±0.88	±0.58	
	MWW	7.31	4.56	4.85	11.16	5.25	6.66	a
		±0.30	±0.28	±0.73	±0.55	±0.39	±0.43	
	b	a	a	c	b	b
Fruit fresh weight (g)	TW	8.93	4.56	3.56	10.63	8.56	7.45	b
		±0.62	±0.45	±0.31	±0.27	±0.18	±0.45	
	DWW	9.77	6.83	5.13	12.60	10.27	9.19	c
		±0.54	±0.31	±0.39	±0.72	±0.56	±0.38	
	LWW	11.34	6.15	5.93	14.33	9.25	10.46	c
		±0.81	±0.31	±0.31	±0.55	±0.42	±0.53	
	MWW	4.27	3.49	3.13	7.75	6.38	7.35	a
		±0.38	±0.34	±0.08	±0.33	±0.52	±0.35	
	b	a	c	b	b
Fruit dry weight (g)	TW	2.22	1.57	1.64	2.82	2.04	1.92	b
		±0.07	±0.09	±0.07	±0.09	±0.12	±0.04	
	DWW	2.86	1.89	1.83	3.44	3.10	3.12	c
		±0.24	±0.04	±0.10	±0.12	±0.11	±0.14	
	LWW	3.12	1.93	1.74	3.34	2.89	2.76	c
		±0.19	±0.07	±0.03	±0.17	±0.15	±0.11	
	MWW	1.54	1.22	1.49	2.25	1.46	1.76	a
		±0.10	±0.09	±0.07	±0.11	±0.04	±0.05	
	b	a	a	c	b	b

TW, tap water; DWW, domestic wastewater; LWW, Lyari wastewater; MWW, Malir wastewater; ±, standard error of replicates; Means followed by letters vary significantly based on Two-Way ANOVA; Tukey's HSD test (*p* < 0.05).

**Figure 3:**
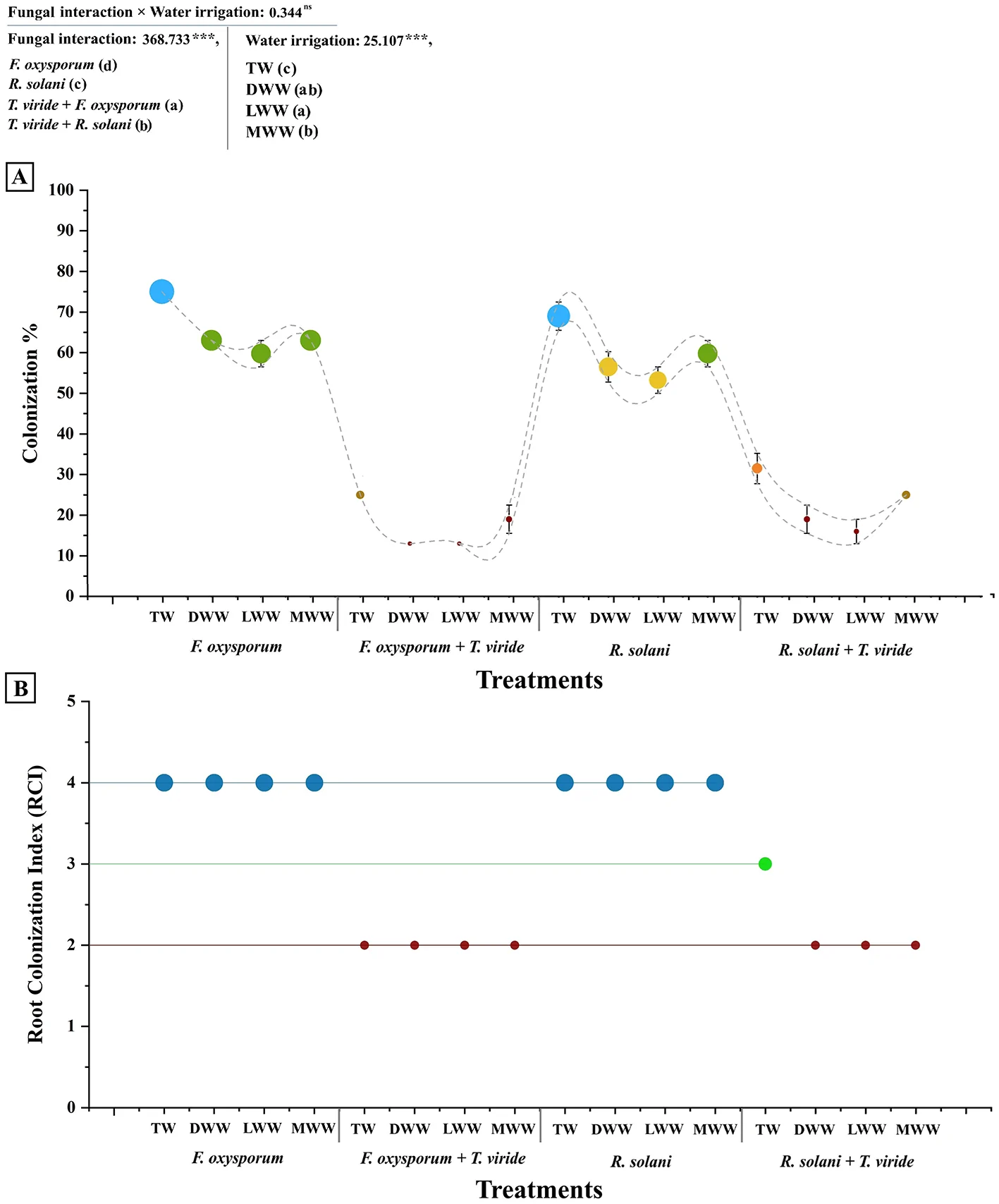
Pathological traits, i.e., **(A)** colonization percentage and **(B)** root colonization index of pathogens in the roots of *Abelmoschus esculentus* (L.) Moench, subjected to various irrigation water qualities with the interaction of the bioprotectant fungus *T. viride*. Means followed by letters vary significantly based on Two-Way ANOVA, Tukey's HSD test (*p* < 0.05).

Artificial infestation with pathogenic fungal species resulted in root rot (Figure 3), leading to a reduction in all growth parameters and plant yield. However, the application of DWW and LWW significantly improved the growth of diseased plants affected by *F. oxysporum* and *R. solani* compared to MWW and control TW irrigation. Additionally, the bioprotectant fungus further stimulated plant growth under pathogenic stress across all water types. Notably, the application of *T. viride* markedly enhanced root length (34.25 ± 1.49 cm), root fresh weight (3.18 ± 0.14 g), root dry weight (2.33 ± 0.09 g), fresh shoot weight (16.58 ± 0.50 g), number of leaves (19.00 ± 1.08), number of fruits per plant (3.50 ± 0.29), and fruit dry weight (2.89 ± 0.15 g) in *F. oxysporum*-infected plants under LWW irrigation. In contrast, shoot length (54.25 ± 1.65 cm), dry shoot weight (4.74 ± 0.19 g), fruit length (11.89 ± 0.84 cm), and fruit fresh weight (10.27 ± 0.56 g) were improved under DWW irrigation. Similarly, *T. viride* enhanced the agronomic traits of *R. solani*-infected plants with LWW irrigation, followed by DWW and TW irrigation.

**Figure 4:**
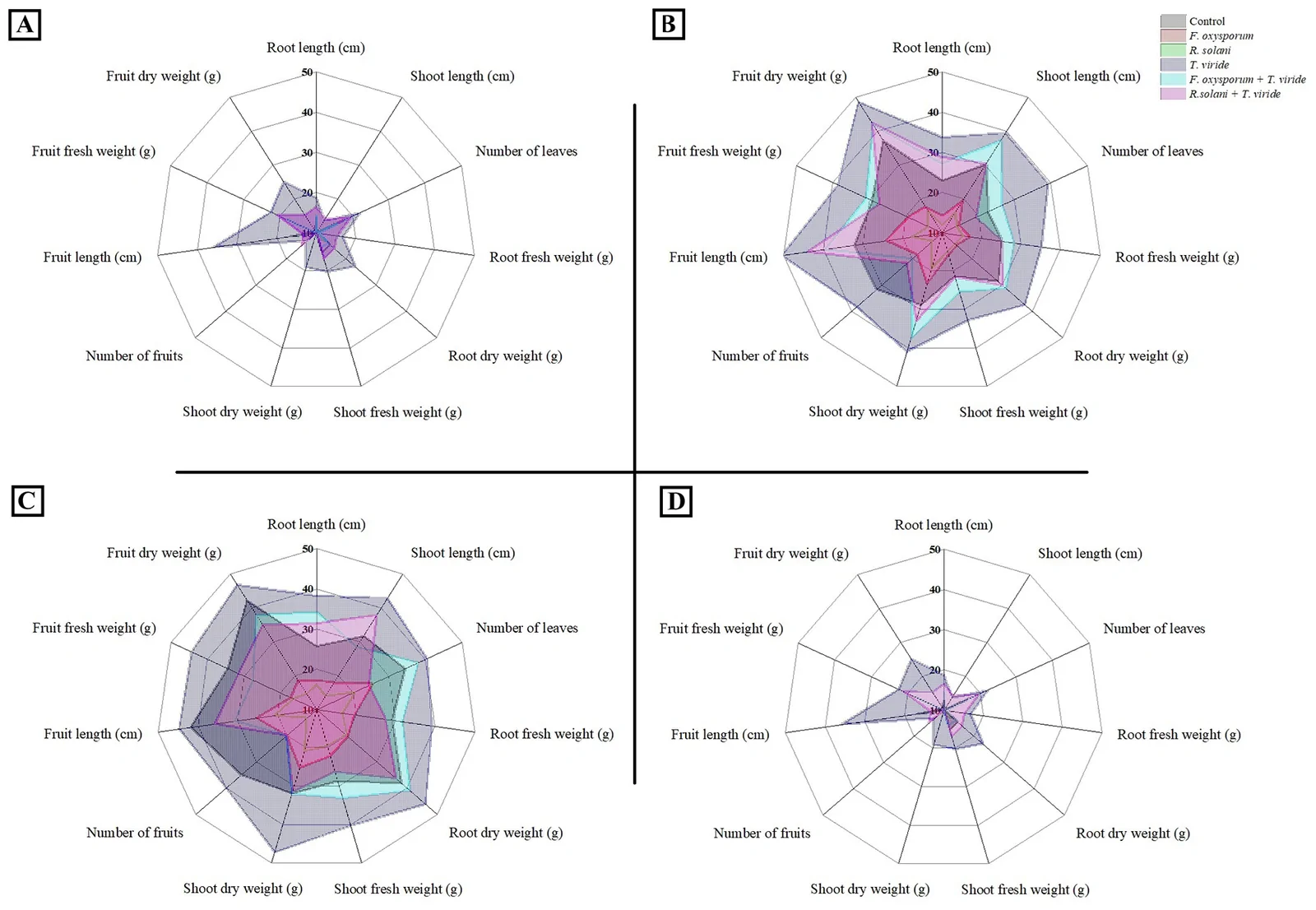
Radar plots illustrating the effects of various irrigation water qualities **(A)** TW, **(B)** DWW, **(C)** LWW, and **(D)** MWW on key plant growth and yield parameters. Each plot compares root and shoot length, weight, and fruit characteristics under control, single fungal inoculation, and co-inoculation treatments. Larger polygon extents indicate stronger overall plant performance. Co-inoculation treatments consistently made the widest profiles, displaying increased plant growth and yield traits relative to single pathogenic inoculations and the control.

The radar plot clearly demonstrated that all DWW and LWW improved agronomic traits compared with the control, with co-inoculation fungal treatments displaying the widest radar profiles (Figure 4). Similarly, irrigations with *T. viride* provided the greatest and most consistent improvements across root and shoot length, weight, and fruit traits, while single inoculations produced moderate but optimistic effects. Overall, the combined treatments revealed a clear synergistic gain, increasing both plant growth and yield traits.

### 3.4 Pathological traits

The colonization percentage of phytopathogenic fungi on *A. esculentus* roots was significantly influenced by various irrigation methods, as illustrated in Figure 3. Both pathogens induced disease in *A. esculentus*, with the highest colonization percentage observed in plant roots irrigated with TW (*F. oxysporum*: 75.00 ± 0.00%, *R. solani*: 69.00 ± 3.46%). In contrast, irrigation with wastewaters, specifically DWW and LWW, reduced colonization percentages by enhancing plant health. The colonization percentage of pathogens in plant roots was further affected by interaction with *T. viride*. It was demonstrated that *T. viride* possesses the ability to reduce pathogen colonization on plant roots across all irrigation types. The most substantial reduction in *R. solani* colonization in plant roots (16.00 ± 3.00%) was achieved by *T. viride* with LWW irrigation, representing a −53% difference compared to TW irrigation without *T. viride* (69.00 ± 3.46%). Similarly, *T. viride* suppressed *F. oxysporum* colonization in plant roots (13.00 ± 0.00%) with LWW and DWW irrigation, showing a 62% difference compared to TW irrigation without *T. viride* (75.00 ± 0.00%). Furthermore, *T. viride* demonstrated efficacy in reducing colonization percentages of both pathogens in plant roots with MWW irrigation. Root colonization percentages were converted to RCI values, as depicted in Figure 4. The highest RCI value for *F. oxysporum-* and *R. solani*-infected plants was obtained without bioprotectant fungus infestation in the soil (4.00). However, a reduced RCI of pathogens in plant roots was observed with the infestation of *T. viride* (2.00), which showed bioprotectant function.

### 3.5 Physiological traits

#### 3.5.1 Determination of photosynthetic pigments in plants

**Figure 5:**
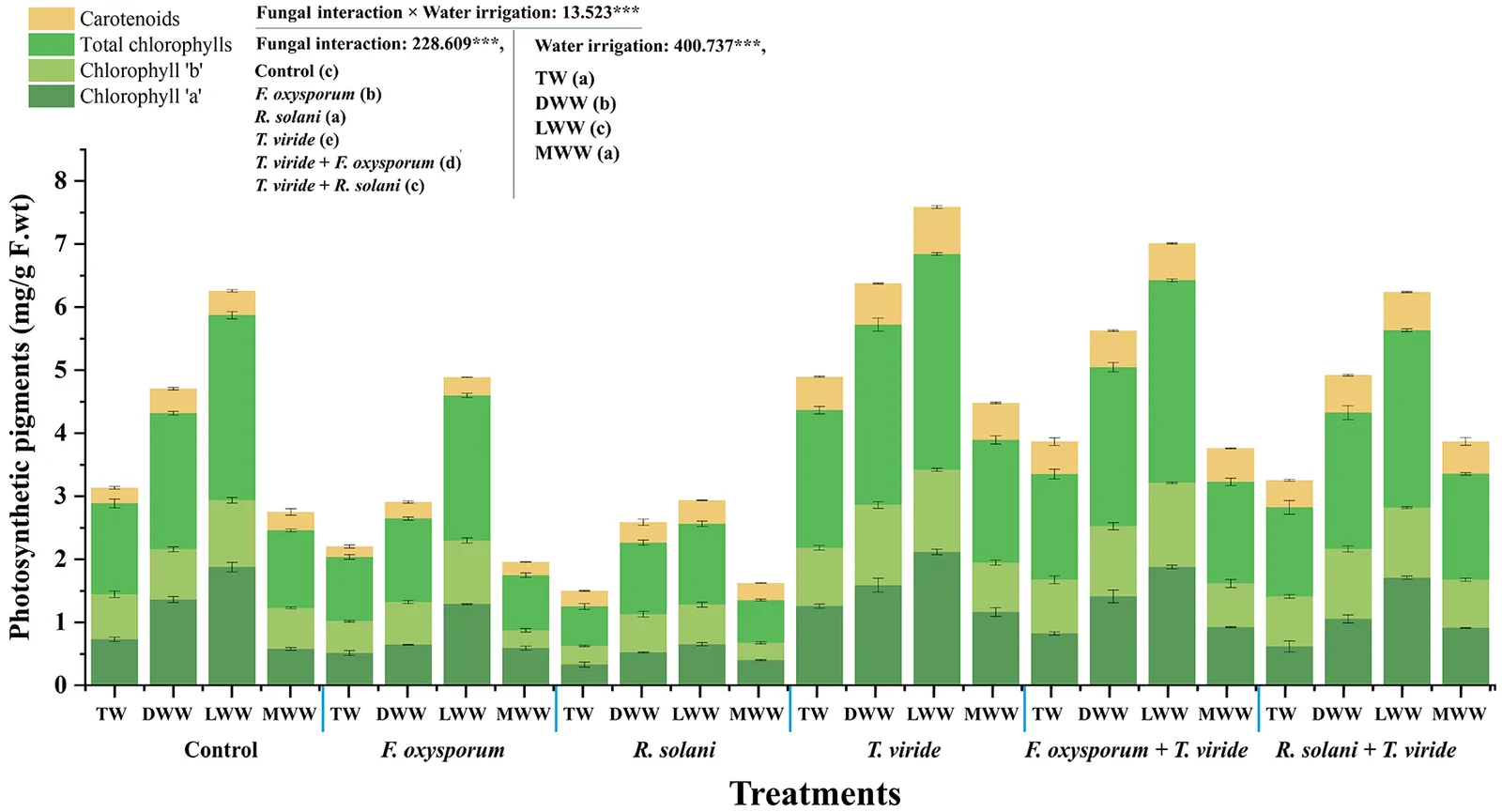
Photosynthetic pigments in *Abelmoschus esculentus* (L.) Moench, subjected to various irrigation water qualities and pathogenic stress with the interaction of the bioprotectant fungus *T. viride*. Means followed by letters vary significantly based on Two-Way ANOVA, Tukey's HSD test (*p* < 0.05).

Irrigation water quality strongly influenced photosynthetic pigment accumulation in plants. Notably, a significant improvement in chlorophyll ‘a' was observed in pathogen-infested plants when *T. viride* was applied with the combination of LWW, reaching 1.88 ± 0.03 mg g^−1^ F.wt in *F. oxysporum*-infected plants and 1.71 ± 0.03 mg g^−1^ F.wt in *R. solani*-infected plants (an increase of +1.37 and +1.38 mg g^−1^ F.wt, respectively), compared with TW-irrigated plants without *T. viride*. Similarly, chlorophyll ‘b' levels in *F. oxysporum*-infested plants increased with LWW treatment and *T. viride* application (1.33 ± 0.01 mg g^−1^ F.wt), representing a +0.82 mg/g F.wt increase. A comparable increase was observed in *R. solani*-infested plants with *T. viride* application and irrigation using DWW and LWW (Figure 5). The total chlorophyll content in LWW-treated plants (*F. oxysporum* 3.21 ± 0.02 mg g^−1^ F.wt, *R. solani* 2.82 ± 0.02 mg g^−1^ F.wt) was +2.19 mg g^−1^ F.wt higher than in pathogen-infected plants. The carotenoid content (0.59 ± 0.01 mg g^−1^ F.wt) was similarly raised in both pathogen-infested plants when *T. viride* was applied with DWW and LWW.

#### 3.5.2 Determination of total proteins

**Figure 6:**
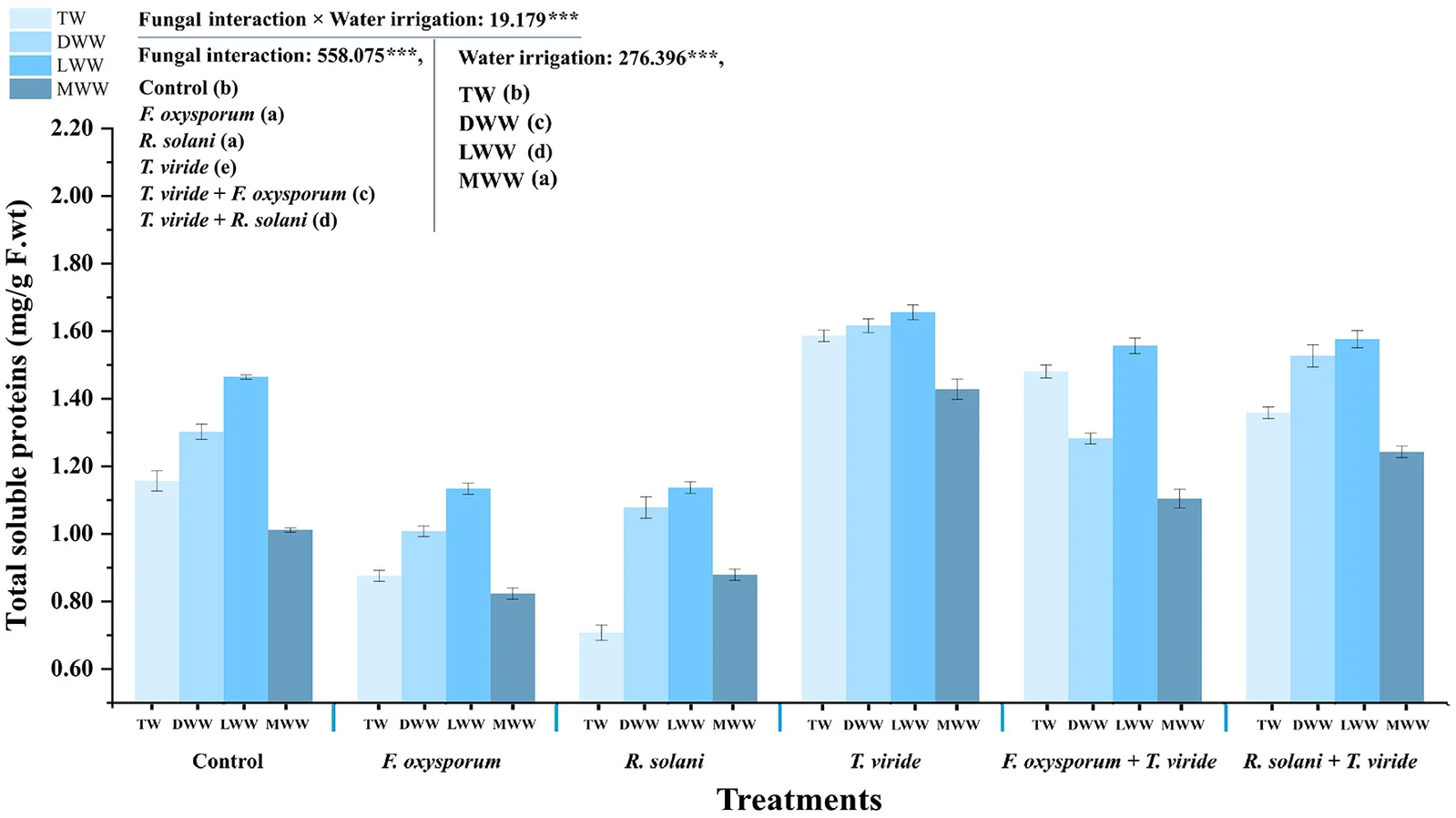
Total soluble proteins in *Abelmoschus esculentus* (L.) Moench, subjected to various irrigation water qualities and pathogenic stress with the interaction of the bioprotectant fungus *T. viride*. Means followed by letters vary significantly based on Two-Way ANOVA, Tukey's HSD test (*p* < 0.05).

The type of water utilized for irrigation significantly influenced the total protein content in plants. The application of LWW (1.46 ± 0.01 mg g^−1^ F.wt) and DWW (1.30 ± 0.02 mg g^−1^ F.wt) markedly increased protein levels compared to plants irrigated with TW (1.16 ± 0.03 mg g^−1^ F.wt). Conversely, the use of MWW resulted in a reduction of protein content (1.01 ± 0.01 mg g^−1^ F.wt). Furthermore, the interaction between fungal species and their combination with water treatments significantly affected total plant protein levels. A reduction in total proteins was observed in plants afflicted by *F. oxysporum* and *R. solani* (Figure 6). However, the infestation by *T. viride* significantly enhanced the total protein content in pathogen-infested plants across all water treatments. The most substantial increase was observed with the combination of *T. viride* and LWW in plants infested with *F. oxysporum* (1.56 ± 0.02 mg g^−1^ F.wt) and *R. solani* (1.58 ± 0.03 mg g^−1^ F.wt), an increase of +0.68 and +0.87 mg g^−1^ F.wt, respectively. Similarly, plants irrigated with DWW exhibited the following results (Figure 6).

#### 3.5.3 Determination of oxidative damage indicators

**Figure 7:**
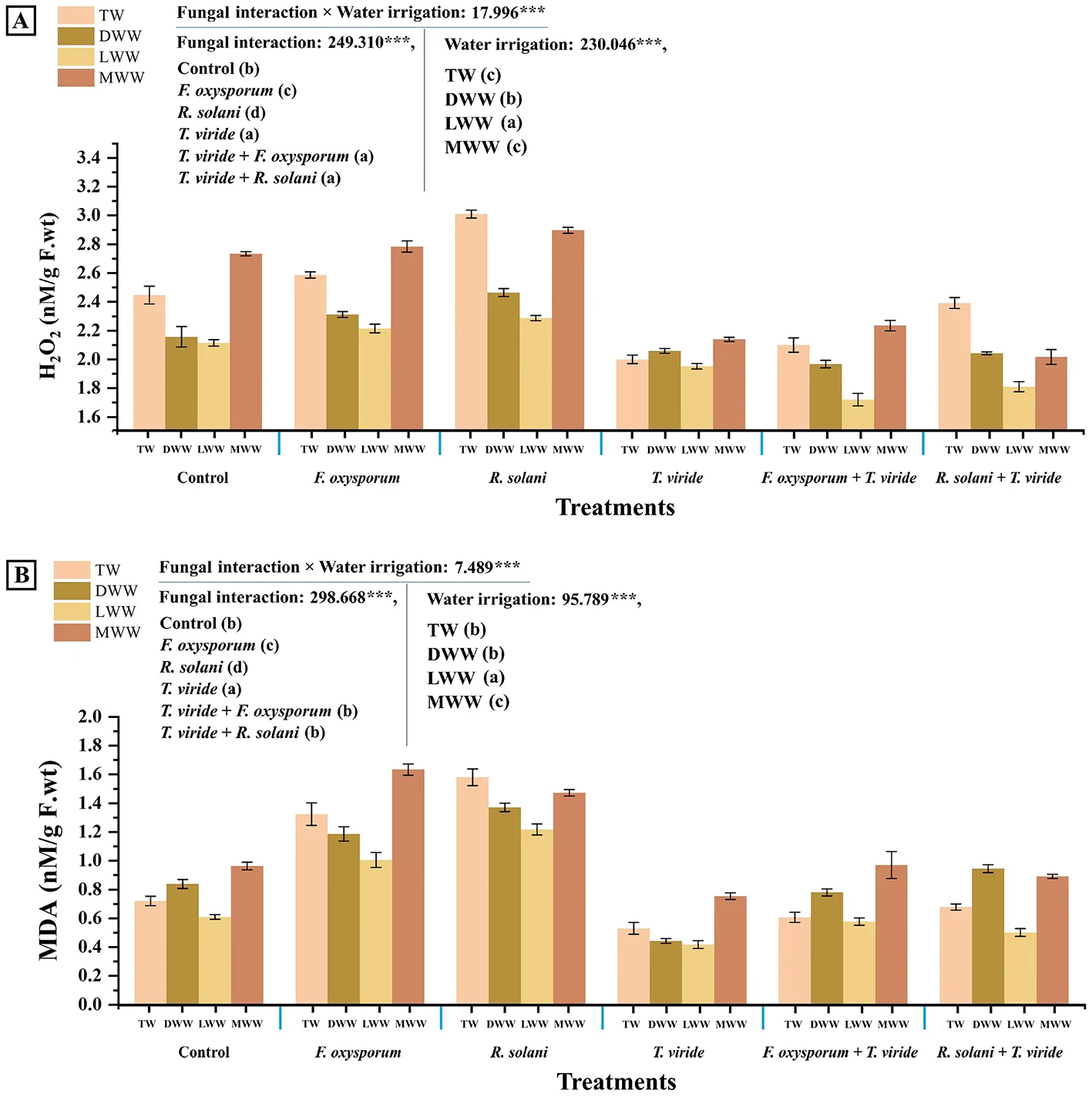
Oxidative damage indicators i.e., **(A)** hydrogen peroxide (H_2_O_2_) and **(B)** malondialdehyde (MDA) in *Abelmoschus esculentus* (L.) Moench, subjected to various irrigation water qualities and pathogenic stress with the interaction of the bioprotectant fungus *T. viride*. Means followed by letters vary significantly based on Two-Way ANOVA, Tukey's HSD test (*p* < 0.05).

The production of stress indicators, specifically hydrogen peroxide (H_2_O_2_) and malondialdehyde (MDA), in plants was significantly influenced by various water treatments. The highest levels of H_2_O_2_ and MDA were observed in plants irrigated with MWW and TW under pathogenic stress conditions (Figure 7). Conversely, the application of *T. viride* with LWW resulted in a reduction in the production of both stress indicators in *F. oxysporum*-infested plants (H_2_O_2_ 1.72 ± 0.04 nm/g F.wt, MDA 0.58 ± 0.03 nm/g F.wt) and *R. solani*-infested plants (H_2_O_2_ 1.81 ± 0.04 nm/g F.wt, MDA 0.50 ± 0.03 nm/g F.wt). This represented a decrease of −0.87 nm/g F.wt (H_2_O_2_) and −0.74 nm/g F.wt (MDA) compared to *F. oxysporum*-infected plants, and −1.20 nm/g F.wt (H_2_O_2_) and −1.08 nm/g F.wt (MDA) compared to *R. solani*-infected plants under TW irrigation without *T. viride* infestation in the soil. The stress-reducing effects of LWW irrigation were followed by those of DWW irrigation.

#### 3.5.4 Activity of antioxidant enzymes

**Figure 8:**
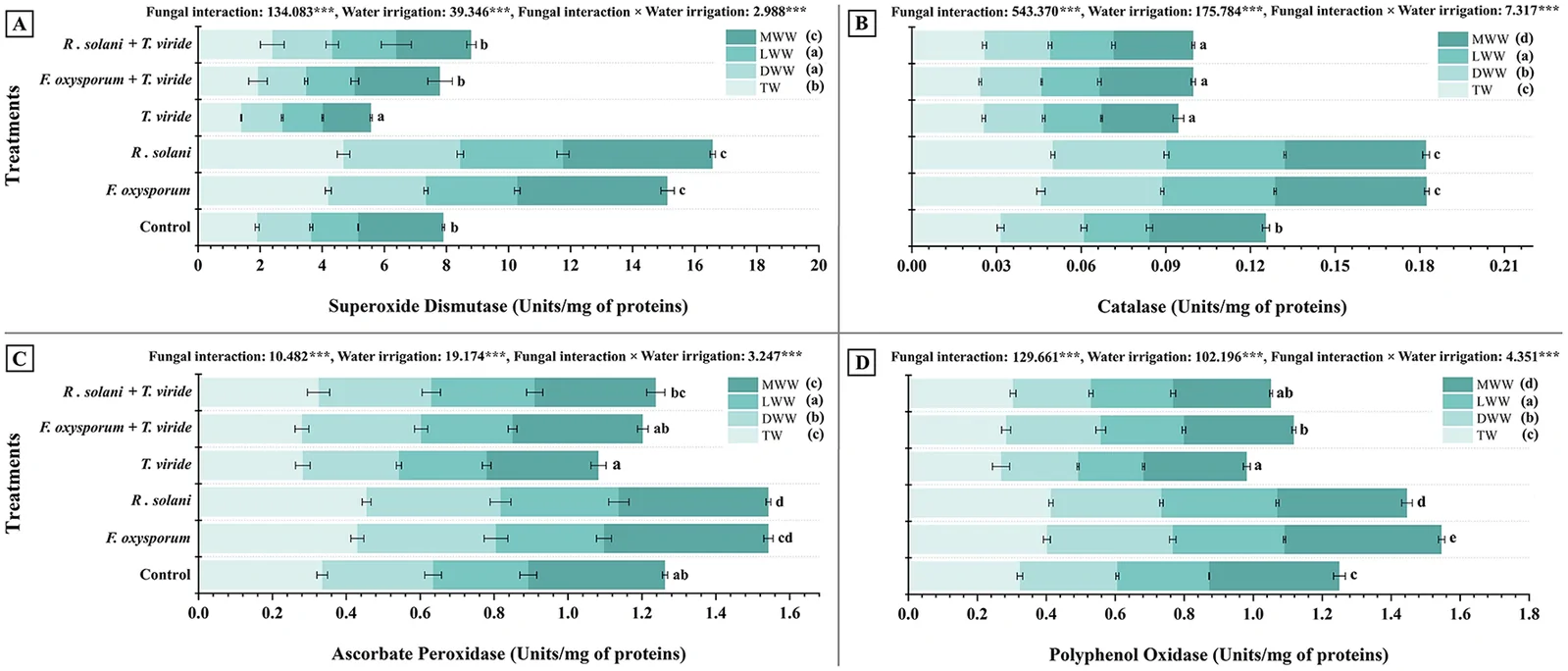
Activity of antioxidant enzymes **(A)** Superoxidase Dismutase, **(B)** Catalase, **(C)** Ascorbate Peroxidase, and **(D)** Polyphenole Oxidase, in *Abelmoschus esculentus* (L.) Moench, subjected to various irrigation water qualities and pathogenic stress with the interaction of the bioprotectant fungus *T. viride*. Means followed by letters vary significantly based on Two-Way ANOVA, Tukey's HSD test (*p* < 0.05).

The application of water of varying qualities significantly influenced the activity of antioxidant enzymes in plants. Plants irrigated with LWW exhibited reduced activity of superoxide dismutase (SOD; 1.51 ± 0.01 units/mg of protein), catalase (CAT; 0.02 ± 0.00 units/mg of protein), ascorbate peroxidase (APX; 0.26 ± 0.02 units/mg of protein), and polyphenol oxidase (PPO; 0.27 ± 0.00 units/mg of protein) compared to those irrigated with control TW. Following LWW, DWW also resulted in decreased antioxidant enzyme activity, whereas MWW irrigation led to increased activity (Figure 8). The interaction between fungal species and water treatments had a highly significant impact on the activity of antioxidant enzymes in plants. Pathogenic species increased the activity of antioxidant enzymes, although DWW and LWW showed comparatively reduced enzyme activity in pathogen-infested plants (Figure 8). However, the inoculation of *T. viride* together with pathogenic species resulted in a reduction of antioxidants across all irrigation types. Among all treatments, *T. viride* with DWW irrigation exhibited the greatest reduction in SOD activity in *F. oxysporum* (1.55 ± 0.05 units/mg of protein) and *R. solani* (1.93 ± 0.20 units/mg of protein) infested plants, followed by LWW irrigation. The activity of CAT (0.02 ± 0.00 units/mg of protein) decreased in both pathogen-infested plants with the application of *T. viride* under DWW and LWW irrigation. The most significant reduction in APX activity was observed in *F. oxysporum* (0.25 ± 0.01 units/mg of protein) and *R. solani* (0.28 ± 0.02 units/mg of protein) infested plants with LWW and *T. viride* application. The activity of PPO in *F. oxysporum-* and *R. solani*-infested plants showed a similar reduction with LWW and *T. viride* application (0.24 ± 0.01 units/mg of protein). Additionally, plants irrigated with DWW also exhibited lower PPO activity in *F. oxysporum* (0.27 ± 0.01 units/mg of protein) and *R. solani* (0.23 ± 0.01 units/mg of protein) infested plants with *T. viride* application.

### 3.6 Correlation analysis

#### 3.6.1 Agronomical traits

**Figure 9:**
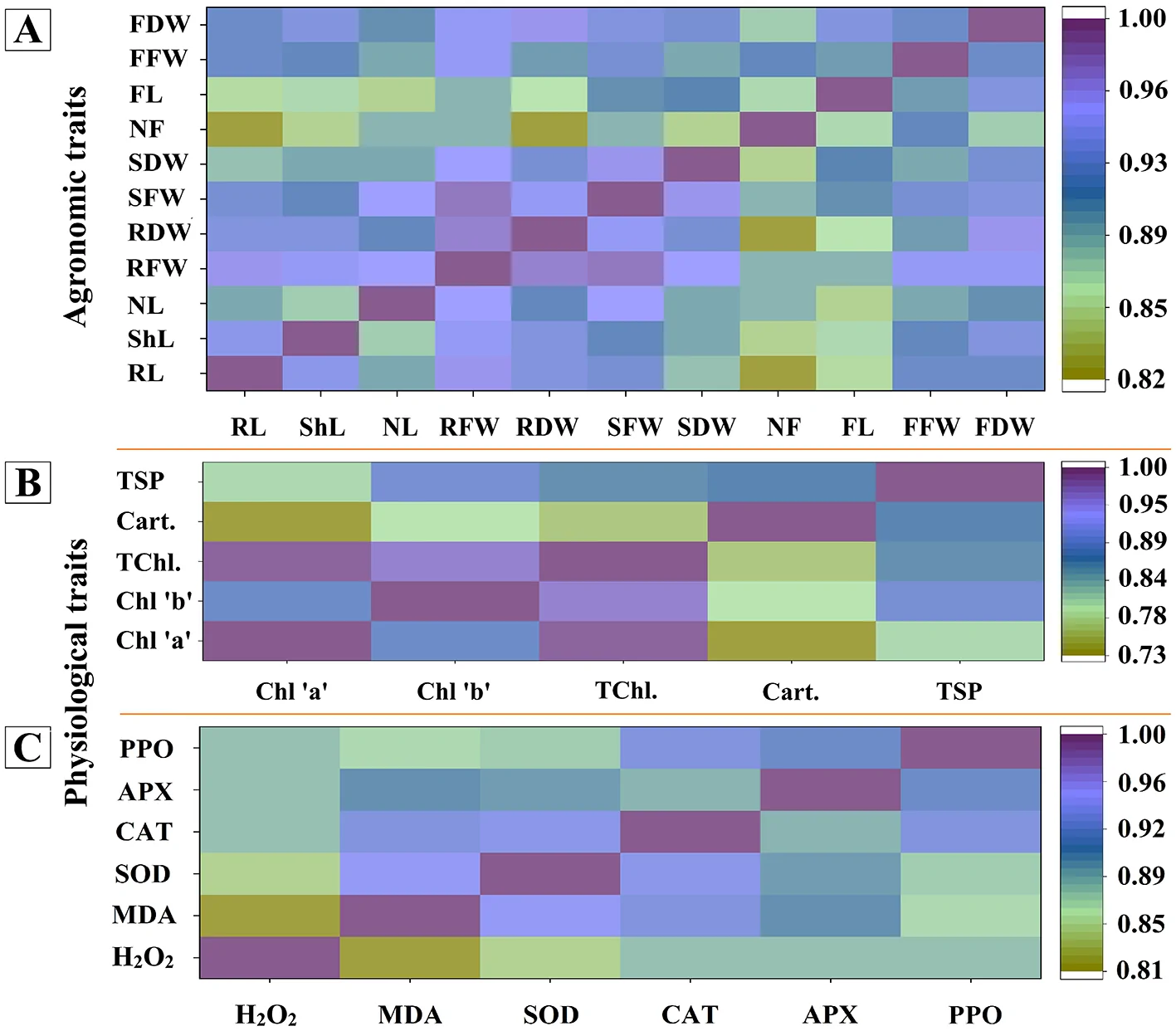
The Pearson correlation coefficient of **(A)** agronomic traits, **(B)** photosynthetic pigments and total soluble proteins and **(C)** oxidative damage indicators and antioxidant enzymes in *Abelmoschus esculentus* (L.) Moench, subjected to various irrigation water qualities and pathogenic stress with the interaction of the bioprotectant fungus *T. viride*. RL, root length; ShL, shoot length; NL, number of leaves; RFW, root fresh weight; RDW, root dry weight; SFW, shoot fresh weight; SDW, shoot dry weight; NF, number of fruits; FL, fruit length; FFW, fruit fresh weight; FDW, fruit dry weight; Chl, chlorophyll; TChl., total chlorophylls; Cart., carotenoids; TSP, total soluble proteins; MDA, malondialdehyde; SOD, superoxide dismutase; CAT, catalase; APX, ascorbate peroxidase; PPO, polyphenol oxidase.

The Pearson correlation coefficient was employed to assess the degree of association between the agronomic data of *A. esculentus* subjected to irrigation with various water types, both with and without the application of biotic stress and the bioprotectant fungus (Figure 9). The analysis revealed highly significant positive correlations among the growth parameters and yield of the plant (*p* < 0.001 for all comparisons). Specifically, root length exhibited a strong correlation with shoot length (*r* = 0.94), root fresh weight (*r* = 0.96), root dry weight (*r* = 0.94), and the number of leaves per plant (*r* = 0.89). Similarly, shoot length demonstrated very high correlations with root fresh weight (*r* = 0.95), shoot dry weight (*r* = 0.89), and the number of leaves per plant (*r* = 0.87). Plant biomass showed the highest degree of interdependence, with root fresh weight strongly correlating with root dry weight (*r* = 0.97) and shoot fresh weight (*r* = 0.98). In parallel, shoot fresh weight was strongly correlated with shoot dry weight (*r* = 0.96) and fruit fresh weight (*r* = 0.93).

The yield of the plant consistently correlated with growth parameters, as evidenced by the positive correlation of fruit number per plant with shoot length (*r* = 0.85), shoot fresh weight (*r* = 0.89), and leaf number (*r* = 0.88). Additionally, fruit length was strongly correlated with shoot dry weight (*r* = 0.91) and fruit dry weight (*r* = 0.93). Fruit fresh weight also exhibited high correlations with root fresh weight (*r* = 0.95), shoot fresh weight (*r* = 0.93), and fruit dry weight (*r* = 0.92). Consequently, the correlation matrix indicates that enhancements in plant growth parameters, such as root and shoot length and biomass, are strongly associated with plant yield. These findings suggest that plant growth allocation serves as a reliable predictor of enhanced plant yield potential.

#### 3.6.2 Plants photosynthetic pigments and total soluble proteins

Pearson correlation analysis demonstrated a robust and significantly positive correlation (*p* < 0.001 for all comparisons) between photosynthetic pigments and total soluble proteins (Figure 9). Within the photosynthetic pigments, chlorophyll a exhibited a strong association with chlorophyll b (*r* = 0.89) and total chlorophylls (*r* = 0.98), thereby confirming the close interdependence of photosynthetic pigments in plants. Additionally, chlorophyll a showed a positive correlation with carotenoids (*r* = 0.73) and total soluble proteins (*r* = 0.81). Chlorophyll b displayed strong and significant correlations with total chlorophylls (*r* = 0.96), carotenoids (*r* = 0.79), and soluble proteins (*r* = 0.89). Total chlorophylls demonstrated the highest correlations with chlorophyll a and b, while also maintaining significant positive associations with carotenoids (*r* = 0.77) and soluble proteins (*r* = 0.86). Similarly, carotenoids were positively correlated with the soluble proteins of plants (*r* = 0.86).

Thus, the correlation matrix indicates a strongly integrated network among photosynthetic pigments and protein contents in plants subjected to various irrigation and fungal treatments. The strong associations between photosynthetic pigments and soluble proteins suggest that enhanced pigment biosynthesis in plants is accompanied by increased protein accumulation. This finding highlights the coordinated regulation of photosynthetic efficiency and metabolic activity in plants under diverse treatments.

#### 3.6.3 Oxidative damage indicators and antioxidant enzymes

The Pearson correlation analysis for oxidative stress-related criteria revealed robust and highly significant positive correlations (*p* < 0.001) among oxidative damage indicators, specifically H_2_O_2_ and MDA, and antioxidant enzymes (SOD, CAT, APX, PPO), as illustrated in Figure 9. The production of H_2_O_2_ in the plant exhibited a positive correlation with MDA (*r* = 0.812), SOD (*r* = 0.844), CAT (*r* = 0.878), APX (*r* = 0.879), and PPO (*r* = 0.878). This finding indicated that the accumulation of oxidative damage indicators was closely linked to the enhanced activity of antioxidant enzymes. Similarly, the lipid peroxidation marker, MDA, demonstrated a very strong association with SOD (*r* = 0.946), CAT (*r* = 0.934), APX (*r* = 0.902), and PPO (*r* = 0.865), suggesting that membrane damage was intricately connected to the plant's defense responses mediated by antioxidant enzymes.

Within the group of enzymes, SOD showed a highly positive correlation with CAT (*r* = 0.943), APX (*r* = 0.897), and PPO (*r* = 0.871). Correspondingly, CAT exhibited a strong association with APX (*r* = 0.885) and PPO (*r* = 0.936). Additionally, APX was strongly correlated with PPO (*r* = 0.920). Thus, the correlation matrix suggests that elevated levels of oxidative damage indicators are consistently accompanied by increased activities of antioxidant enzymes, highlighting the integrated role of these enzymes in mitigating oxidative damage.

The correlation analysis delivered a mechanistic vision into how plants coordinated their oxidative stress responses to the provided treatments. Robust positive associations between H_2_O_2_ and MDA with all antioxidant enzymes (SOD, CAT, APX, PPO) demonstrate that rises in oxidative damage were strongly unified with the activation of enzymatic defense mechanisms. For instance, H_2_O_2_ exhibited high correlations with CAT (*r* = 0.878) and APX (*r* = 0.879), signifying that plants suffering from more ROS accumulation simultaneously increased both H_2_O_2_-scavenging routes. Similarly, the very strong correlations between MDA and SOD (*r* = 0.946) and CAT (*r* = 0.934) reveal a coordinated response, in which membrane lipid peroxidation triggered increased ROS detoxification extent.

Significantly, the strong enzyme interrelationships, e.g., SOD/CAT, *r* = 0.943; CAT/PPO, *r* = 0.936, indicate that antioxidant enzymes did not perform independently but shaped an integrated defense network. This trend reinforced the interpretation that the treatment-driven mitigation of oxidative stress was achieved through a coordinated enzymatic performance rather than isolated variations in individual enzymes. Thus, the correlation matrix revealed functional coupling among oxidative damage indicators and antioxidant enzymes, supporting the mechanistic clarification of how the treatments counteracted stress.

### 3.7 Hierarchical clustering

#### 3.7.1 Agronomical traits

**Figure 10:**
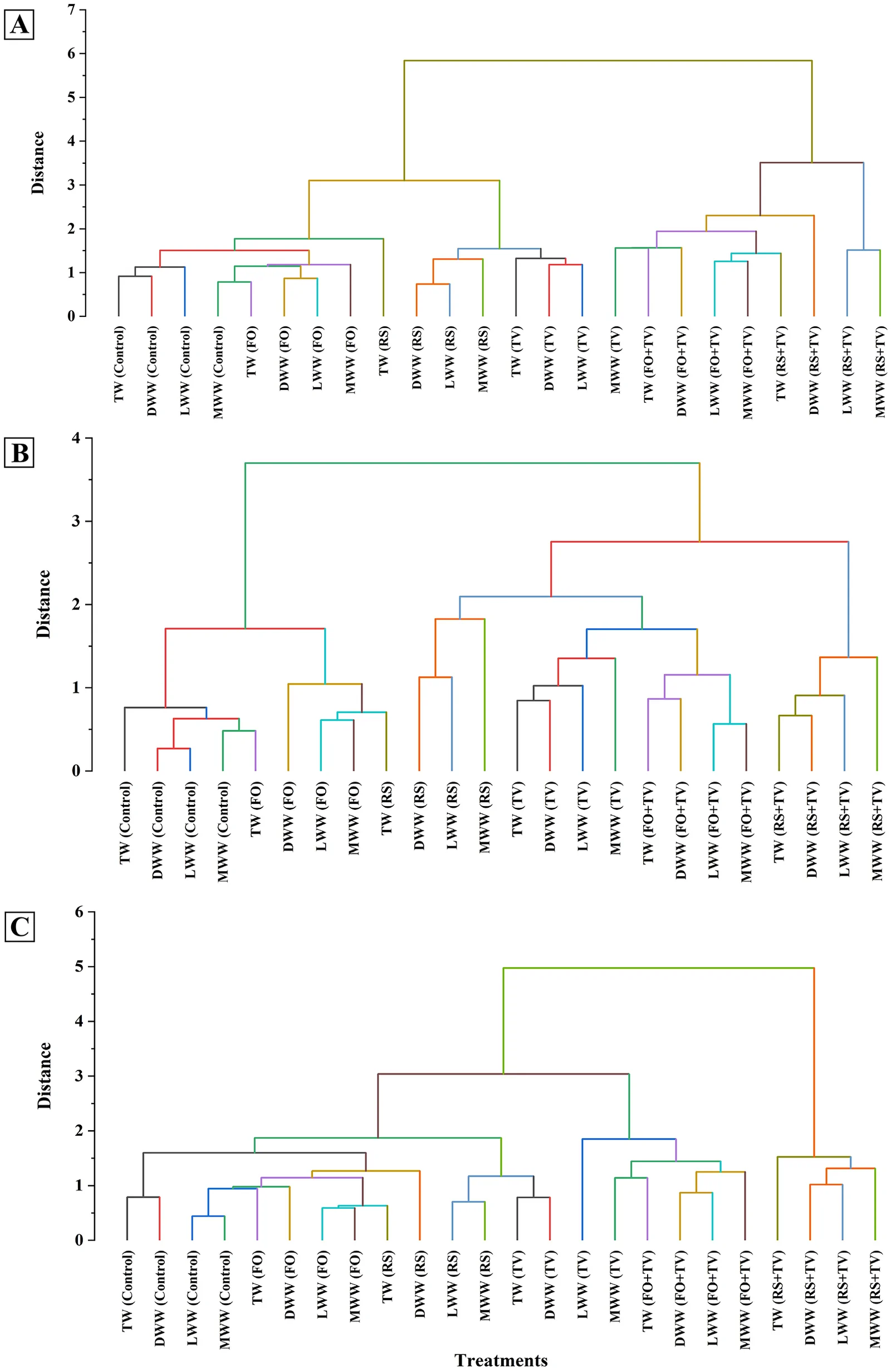
The Hierarchical clustering using Ward's linkage and Euclidean distance of **(A)** agronomic traits, **(B)** photosynthetic pigments and total soluble proteins and **(C)** oxidative damage indicators and antioxidant enzymes in *Abelmoschus esculentus* (L.) Moench, subjected to various irrigation water qualities and pathogenic stress with the interaction of the bioprotectant fungus *T. viride*.

Hierarchical clustering was conducted to assess the similarities among 24 treatment combinations, which encompass various water irrigation methods and fungal applications on the agronomic traits of plants (Figure 10). The dendrogram, which group treatments based on the overall response profiles of plants, reveals distinct clusters that highlight underlying agronomic traits. The dendrogram distinctly separates the non-fungal infestation with TW, DWW, LWW, MWW, from those involving fungal applications, indicating a clear divergence in plant growth and yield parameters. Treatments such as *F. oxysporum* (FO) + *T. viride* (TV) and *R. solani* (RS) + TV formed highly compact clusters, signifying the synergistic influence of mutual fungal interactions. Notably, TW (FO + TV) and TW (RS + TV) are closely clustered, suggesting that TW irrigation responded similarly to both pathogens in conjunction with the interaction of *T. viride*. Wastewater irrigations, namely DWW, LWW, and MWW, exhibited more discrete clustering, particularly with pathogens and *T. viride* applications, indicating variable responses dependent on water quality and fungal species. However, the agronomic responses of LWW and DWW with pathogens and bioprotectant fungus interactions clustered together, demonstrating comparable stress mitigation and growth improvement under combined treatment.

#### 3.7.2 Plants photosynthetic pigments and total soluble proteins

Hierarchical clustering was employed to evaluate distinct clustering patterns of soluble proteins and plant pigments, thereby revealing treatment-specific physiological responses in plants, including photosynthetic pigments and total soluble proteins (Figure 10). Non-fungal infestation with TW, DWW, LWW, and MWW formed a distinct cluster, indicating standard similarity in the absence of fungal infection across irrigation types. Treatments involving single fungal species, namely *F. oxysporum, R. solani*, or *T. viride*, demonstrated significant divergence, with TW clustering more robustly than wastewater irrigations, which exhibited greater variability.

Notably, the interaction of pathogens with *T. viride* resulted in well-defined clusters across all irrigation types. TW in combination with FO + TV and RS + TV, formed strong clusters, indicating similar enhancements in photosynthetic pigments and total soluble proteins in plants irrigated with TW. In contrast, LWW and DWW, when combined with pathogens and *T. viride*, displayed co-localized grouping, suggesting equivalent stress mitigation, as evidenced by robust photosynthetic pigments and total soluble proteins.

#### 3.7.3 Oxidative damage indicators and antioxidant enzymes

The subsequent hierarchical clustering analysis of oxidative stress-related factors provided evidence of clustering patterns that reflect the responses of oxidative damage indicators and antioxidant enzymes (Figure 10). Plant irrigations using TW, DWW, LWW, and MWW, in the absence of fungal species, formed a distinct cluster, indicating a standard similarity in the responses of oxidative damage indicators and antioxidant enzymes. While individual fungal species exhibited notable differences, the TW clustering was more closely related than the wastewater irrigations, which demonstrated further variations.

Conversely, the interaction of pathogens with *T. viride* resulted in clearly defined clusters across all irrigation types. For instance, TW with FO + TV and RS + TV were compactly grouped, indicating similar effects on plant response. LWW and DWW, when combined with pathogens and *T. viride*, clustered together, revealing equivalent responses in oxidative stress and mitigation through antioxidant enzyme activity in plants.

These findings underscore the utility of hierarchical clustering in identifying treatment similarities, demonstrating that the combined water irrigations, particularly DWW and LWW, with *T. viride*, enhance plant growth parameters, yield, and physiological activities, even under pathogenic stress.

## 4 Discussion

### 4.1 Effect of wastewaters on plant growth and yield

Wastewater serves as a valuable source of liquid fertilizer for crops (Karef et al., 2025). However, the suitability of wastewater for plant use is contingent upon the quality that plants can tolerate, which is influenced by the sources from which the wastewater is discharged. In the present study, wastewaters with varying quality parameters exhibited distinct yet highly significant effects on plant growth and yield. Elevated levels of physicochemical parameters and heavy metals and toxic metalloids such as Cd and As, exceeding WHO standards (WHO, 2006) in MWW, adversely impacted plant biomass, length, and yield (Hassan et al., 2022; Shah et al., 2022). The excessive uptake of these elements by plants disrupts metabolic activities and biosynthesis within the plant body (Fakhar et al., 2022), leading to stunted growth and development, as well as a decline in the quality and quantity of the total plant yield (Wen et al., 2021). Conversely, LWW and DWW contained higher concentrations of essential nutrients that enhanced plant growth and productivity. Notably, previous studies reported that wastewaters contribute to the enhancement of soil microbial diversity, enriching fungi and various bacterial groups, including actinomycetes, and bacteria, thereby improving and facilitating the soil nutrient availability for plant growth (Shah et al., 2024; Paliaga et al., 2025). In this context, Abou Jaoude et al. (2025) observed an increase in essential soil nutrients through wastewater irrigation, which improved the growth of *Solanum tuberosum* and *Zea mays*.

Conversely, while various chemical and physical mechanisms exist for the removal of heavy metals from water and soil, these methods are often sophisticated and costly. Biological approaches, however, present a viable alternative for addressing this issue (Oziegbe et al., 2025). One such approach involves the utilization of microorganisms, which have a significant capacity to absorb heavy metals (Wei et al., 2025). These microbes also play a crucial role in enhancing plant growth, thereby serving as substitutes for synthetic fertilizers that can lead to the accumulation of toxic chemicals in plants and soil (Adetunji et al., 2022; Basheer et al., 2023). Similarly, Liaquat et al. (2020) identified *A. aculeatus, A. sclerotiorum, A. niger, Komagataella phaffii*, and *T. harzianum* as having tolerance to Cd, Cr, and Pb. Current findings indicate that *A. esculentus* (L.) Moench, when irrigated with wastewater, demonstrated improved growth parameters and yield following the application of *T. viride* (PZ212855) in the soil. Among the various irrigation methods, LWW showed the greatest enhancement in plant growth and yield when combined with the bioprotectant fungus. Additionally, DWW also resulted in better growth following LWW irrigation. In a similar direction, researchers reported a reduction of heavy metals in a liquid medium of 100 mg L^−1^, such as Pb, Cd, As, Zn, and Cu, through the application of *A. niger, A. flavus*, and *T. viride*. They also observed significant improvements (upto 40%) in maize, wheat, barley, and sorghum growth parameters by introducing the bioprotectant fungus into heavy metal-contaminated soil (Dejene et al., 2026; Metwally and Abdelhameed, 2026). *T. viride* is renowned for enhancing plant growth due to its ability to secrete phosphate-solubilizing enzymes, phytohormones, and siderophores (Ferreira et al., 2024). Consequently, various researchers have demonstrated *T. viride*'s efficacy as a growth promoter for plants such as melon and cucumber (Lian et al., 2023; Liu et al., 2025), owing to its capacity to decompose organic matter and make nutrients available to plants (Abubakar et al., 2025). *T. viride* also possesses the ability to accumulate heavy metals and remove them from the ecosystem. Its cell wall contains chitin and glucan polymers that bind heavy metals, such as Cd (Złotko et al., 2019). According to Metwally and Abdelhameed (2026), *T. viride* has the potential to absorb Cd, exhibiting growth in a medium containing Cd at concentrations tolerated up to 200 ppm, while simultaneously alleviating Cd toxicity in wheat plants. Thus, consistent with physicochemical and elemental analysis of the wastewater, *T. viride* application improved plant growth even under elevated stress conditions in the current study.

### 4.2 Pathogenicity

Growing plants in soil inoculated with pathogens exhibited various symptoms, including chlorosis, necrosis, and wilting, due to the infection of plant roots, which resulted in stunted plant growth. The application of *T. viride* in soil, combined with irrigation using wastewater under biotic stress, demonstrated improved growth parameters and yield. However, as noted by various researchers, fluctuations in environmental factors influence the production rate of secondary metabolites by protective microbes against pathogens (Baazeem et al., 2021). *In vivo* pathogenicity tests of *F. oxysporum* and *R. solani* were conducted to assess the colonization percentage in plant roots. Both inoculums were confirmed as pathogens, causing diseases in plants. The colonization percentage of phytopathogenic fungi on plant roots was significantly affected by different types of water irrigation. According to Zhao et al. (2021), *R. solani* also causes sheath blight in rice, which is challenging to manage. Meanwhile, some researchers have reported the severity of wilt and root rot diseases in wheat and barley varieties grown in *F. falciforme*-infested soil (Qostal et al., 2025). In the current study, plants grown in *R. solani* and *F. oxysporum*-inoculated soils exhibited symptoms of root rot, leading to wilt, chlorosis, and necrosis.

Conversely, the colonization percentage of pathogens was significantly influenced by the interaction with the bioprotectant fungus *T. viride*. The greatest reduction in colonization of both pathogens was observed with the cocolonization with *T. viride* and LWW irrigation. Following LWW, DWW irrigation also demonstrated substantial reductions in pathogen colonization percentages. Similarly, El Kazzaz et al. (2022) reported a higher reduction in root colonization of pathogens causing root rot and wilt diseases, specifically *F. oxysporum* f. sp. *capsici* and *R. solani* in pepper plants, through the application of *Paenibacillus polymyxa* and *T. longibrachiatum*. This is attributed to the fungal pathogenic cell wall, which comprises chitin, glucan, and proteins. Trichoderma sp. secretes several lytic enzymes, including β-1,3 glucanases, chitinases, and proteases, to hydrolyze these components of the cell wall of the pathogens (Brazhnikova et al., 2025). The present study concurs with these findings, demonstrating that *T. viride* can control pathogenic invasion of plant roots through colonization, resulting in significantly improved growth and increased yield with DWW and LWW irrigation compared to the control (Shah et al., 2020, 2022).

### 4.3 Integrated plant physiological responses

#### 4.3.1 Photosynthetic pigments

Recent studies have indicated that wastewater can enhance the levels of photosynthetic pigments, such as total chlorophylls and carotenoids, in *Moringa oleifera*, in contrast to wastewater rich in heavy metals (Melo et al., 2025). Similarly, current research has demonstrated an increase in photosynthetic pigments in *A. esculentus* when irrigated with DWW and LWW, whereas MWW was found to reduce these pigments in plant leaves with elevated concentrations of As and Cd. The increased levels of photosynthetic pigments in plants may be attributed to the higher content of organic matter and essential nutrients present in the irrigated water (Rekaby et al., 2021). Consistent findings have been reported by other researchers, who observed enhanced photosynthetic content and improved yield with high nutritional value in *Hordeum vulgare* (Alvarez Holguin et al., 2022). Photosynthetic pigments are crucial for plant growth, with carotenoids functioning as non-enzymatic antioxidants that protect plants from oxidative stress by acting as electron donors and reducing ROS to less harmful molecules (Taïbi et al., 2025). Conversely, current studies have shown a decrease in photosynthetic pigments in plant leaves under biotic stress. Pathogens such as *F. oxysporum* and *R. solani* obstruct water and nutrient uptake channels in plant roots by causing root infections with high colonization percentages. Lu et al. (2025) also noted that the biosynthesis of photosynthetic pigments is affected by an imbalance of essential nutrients within the plant. Furthermore, it has been reported that the reduction in total chlorophylls and carotenoids in plants is due to heavy metal-contaminated soil, which interferes with chlorophyllase activity, chloroplast deformation, and the stability of protein-related pigment complexes (Su et al., 2025). A significant reduction in photosynthetic pigments has been observed when plants are irrigated with MWW under biotic stress. Consequently, the increased biomass and yield in plants can be attributed to the higher levels of photosynthetic pigments in leaves (Ferreira et al., 2025). The presence of bioprotectant fungus in the soil has been shown to further enhance photosynthetic pigment levels.

#### 4.3.2 Oxidative damage indicators

Hydrogen peroxide (H_2_O_2_) is a reactive oxygen species (ROS) generated in response to both abiotic and biotic stressors (Dumanović et al., 2021). It is identified as a contributor to lipid peroxidation, like various other ROS, such as hydroxyl radicals (OH^•^), singlet oxygen (^1^O_2_), and superoxide anions (•${\text{O}}_{2}^{-}$) (Garcia Caparros et al., 2021). When lipid peroxidation occurs in plant leaves, it results into the production of malondialdehyde (MDA) which is recognized as its terminal output (Chrysargyris and Tzortzakis, 2025). Recent studies have similarly demonstrated an increase in MDA levels concomitant with elevated H_2_O_2_ production in the leaves of *A. esculentus* under pathogenic stress. The stress was observed to double when the plants were irrigated with MWW. These observations suggest that oxidative stress in plants, induced by MDA and H_2_O_2_, leads to disrupting metabolic processes, thereby causing cellular damage at accumulation sites (Rao et al., 2025). Similarly, infection by *Pseudomonas syringae* pv. *syringae* triggers a hypersensitive response in plants, marked by increased superoxide (•${\text{O}}_{2}^{-}$) production and lipid peroxidation (Stahl et al., 2019; Adigun et al., 2021). The foliar application of *T. viride* was shown to decrease H_2_O_2_ levels, membrane ion leakage, and MDA in tomato plants by suppressing early blight disease, ultimately enhancing plant biomass and yield (Khalil et al., 2021). In the current findings, treatment with bioprotectant fungus reduced the production of MDA and H_2_O_2_ by inhibiting the mycelial growth of pathogens, which resulted in the lowest root colonization percentage by pathogens. Furthermore, irrigation of diseased plants with LWW and DWW enhanced the activity of bioprotectant fungus, thereby inhibiting H_2_O_2_ production in the plants.

#### 4.3.3 Total soluble proteins

The proliferation of photosynthetic pigments in plants was associated with increased total soluble protein when the soil was inoculated with the heavy-metal-tolerant, plant-growth-promoting fungus *T. harzianum*, compared with control plants (Kamal et al., 2025). Similarly, irrigation with LWW and DWW resulted in elevated levels of total proteins alongside an increase in photosynthetic pigments. Both types of wastewaters contained higher concentrations of essential nutrients necessary for protein synthesis in plants. Conversely, a reduction in protein content was observed under pathogenic stress, which obstructed nutrient uptake by colonizing the roots. However, the bioprotectant fungus improved plant health by inhibiting pathogenic growth in the rhizosphere, thereby enhancing total protein levels in the plant. Metwally et al. (2021) similarly reported an increase in soluble proteins in *Allium cepa* following treatment with *T. viride*, which also led to improved crop production. In contrast, Kaczur et al. (2025) documented a reduction in total proteins and photosynthetic pigments in *Zea mays* when irrigated with wastewater containing high concentrations of heavy metals, ultimately leading to decreased yield production. The present findings align with this report, as irrigation with MWW reduced protein levels in *A. esculentus*, resulting in diminished yield production.

#### 4.3.4 Antioxidant enzymes

Heavy metals like Cd and As exert phytotoxic effects mainly through ROS overproduction that leads to lipid peroxidation, macromolecular degradation, disrupts nutrient uptake, and reduced plant growth (Teng et al., 2025; Geng et al., 2024). Although, antioxidant enzymes such as catalases and peroxidases play a crucial role in the elimination of ROS, including H_2_O_2_, OH•, and •${\text{O}}_{2}^{-}$, from the plant body (Sami et al., 2021). The enhancement of antioxidants is a natural defense mechanism in plants to overcome stress by reducing the osmotic and toxic effects of ROS through scavenging (Sitara et al., 2023; Xing et al., 2025). Similarly, current findings related to toxicity indicators in the form of H_2_O_2_ and MDA correspond to a reduction in plant growth parameters. To cope with stress, plants exposed to higher concentrations of As and Cd exhibit the highest activity of antioxidant enzymes, such as SOD, CAT, and APX. Likewise, ALKahtani et al. (2020) reported significantly higher antioxidant concentrations in plants under salinity stress. For the reason that, antioxidant enzymes such as SOD play a crucial role in oxidative metabolism by converting the superoxide radical into H_2_O_2_ (Batool et al., 2022). CAT is primarily observed in peroxisomes and converts H_2_O_2_ into water molecules. APX is responsible for detoxifying H_2_O_2_ generated by SOD in chloroplasts through the conversion of superoxide ions during photosynthesis (Molina Moya et al., 2025).

Antioxidant activity was further enhanced under pathogenic stress induced by *F. oxysporum* and *R. solani*. Conversely, plants treated by growth-promoting rhizobacteria boosted plant resistance and mitigated the activity of antioxidant enzymes, even under salinity stress (Chen et al., 2025b). This is because antioxidants are produced as needed to neutralize toxic oxygen species in plants (ALKahtani et al., 2020; Sitara et al., 2023). Correspondingly, the presence of bioprotectants in the soil led to a reduction in antioxidant activity by inhibiting the mycelial growth of pathogens. Furthermore, the combination of bioprotectant fungus with the application of LWW and DWW further alleviated stress in the plant body. Consequently, the present findings are consistent with the results reported by Zapata Sarmiento et al. (2025).

Consequently, the decrease in MDA and H_2_O_2_ detected in *T. viride*-treated plants is consistent with its well-established activity of bioprotectant against soil-borne pathogens. *T. viride* suppresses the mycelial growth of *F. oxysporum* and *R. solani* through mycoparasitism, antibiosis, and secretion of cell wall-degrading enzymes (Shah et al., 2020; Huang et al., 2022; Natsiopoulos et al., 2022). This suppression of pathogen growth results in reduced root colonization, which in turn decreases pathogen-induced oxidative bursts, typically associated with raised H_2_O_2_ and lipid peroxidation (Metwally and Abdelhameed, 2026). Moreover, *T. viride* improves the plant's antioxidant mechanism, further lessening ROS accumulation (Rai and Solanki, 2023). Together, these mechanisms elucidate the lower MDA and H_2_O_2_ production observed in the bioprotectant-treated plants.

Current studies have revealed varied consequences when wastewater irrigation is integrated with *Trichoderma* spp. inoculation. Trotta et al. (2024) described that *T. afroharzianum* improved antioxidant defenses in *Solanum lycopersicum* under reclaimed wastewater, but did not significantly increase morphological characteristics. Similarly, *T. harzianum* has been found to increase *Lactuca sativa* biomass and impact contaminant dynamics under wastewater irrigation, demonstrating a contribution to growth promotion and soil detoxification (Brienza et al., 2026). By comparison, the present study demonstrates that *T. viride* not only mitigates heavy metal-induced oxidative stress but also noticeably improves agronomic traits and yield of *A. esculentus* under dual biotic and abiotic stress. This suggests that previous findings emphasized either growth stimulation or stress regulation. However, the present outcomes provide novel evidence of a synergistic effect, where wastewater nutrients and fungal bioprotection interactively improve crop resilience and productivity.

Although LWW and DWW had analogous nutrient compositions, plants irrigated with LWW constantly showed robust growth and yield production. This variance potentially stems from synergistic microbial interactions present in LWW (Shah et al., 2024), which could have enabled nutrient mineralization, promoted rhizosphere activity, and improved plant absorption efficiency. Earlier studies have revealed that wastewater-associated microbial consortia can accelerate plant growth by secreting siderophores, phytohormones, and enzymes that mobilize nutrients while concurrently conquering pathogens (Marra et al., 2021; Rai and Solanki, 2023). Conversely, the slightly higher arsenic level found in DWW (0.185 mg L^−1^ ± 0.002) may have induced further oxidative stress, limiting the functional benefits of its nutrient abundance. Therefore, the improved performance under LWW irrigation likely reveals its nutrient composition and also the contribution of useful microbial consortia that interact positively with *T. viride*, increasing its bioprotective impacts. Whereas arsenic-loaded DWW restricted its comparative efficiency. However, while wastewater reuse delivers agronomic advantages, its safe and sustainable implementation requires complementary biological approaches, like microbial bioprotectants, to mitigate associated risks.

Thus, wastewater irrigation offers essential nutrients but also introduces stresses from toxic elements, i.e., arsenic. However, recent investigations have validated that *T. viride* has the potential to mitigate these stresses through several mechanisms. Such as, the cell wall of *T. viride* acts as a biosorbent, binding and sequestering heavy metals like As, thereby decreasing their bioavailability to plants (Chen et al., 2025a; Shao et al., 2025). Besides, this bioprotectant also secretes organic acids and chelating metabolites that hold As and other toxic elements, restraining their uptake in plants. Furthermore, *T. viride* improves plant antioxidant defense through enhancing enzyme expression, which attenuates oxidative damage driven by wastewater contaminants and arsenic toxicity (Basheer et al., 2022; Gao et al., 2022). The bioprotectant *T. viride* also suppresses soil-borne plant pathogens through the production of glucanases, chitinases, and antimicrobial metabolites, thereby lessening biotic stress (Vicente et al., 2022; Natsiopoulos et al., 2022). Overall, these complementary mechanisms elucidate the observed reduction in oxidative damage and enhanced growth of plants irrigated with As-laden wastewater, indicating the potential of *T. viride* to ease wastewater-induced stress and enable nutrient-loaded irrigation sources to be exploited as feasible inputs for sustainable agricultural production.

### 4.4 Practical feasibility and applied relevance

Beyond the agronomic and physiological advantages demonstrated in the present study, the practical feasibility of wastewater irrigation integrated with *T. viride* requires careful assessment before endorsing field-level implementation. Cost-benefit investigations from earlier wastewater irrigation studies indicate significant savings in fertilizer consumption, as nutrient-loaded wastewater lessens reliance on synthetic chemical fertilizers. But the costs associated with wastewater monitoring and fungal inoculum production should be incorporated into economic evaluations. However, the economic benefit can increase farmer adoption, mainly in resource-constrained contexts. However, farmer adoption also requires addressing several challenges, like awareness, training, and willingness to integrate bioprotectants into existing practices, which vary extensively across regions. Without passable extension facilities and demonstration trials, application may remain inadequate. Nevertheless, adoption relies on regulatory regimes that facilitate adherence to wastewater quality standards, ensure consumer well-being, and safeguard the environment. Therefore, clear guidelines on permissible contaminant limits, monitoring practices, and certifications are vital to ensure safe and sustainable usage. In regions that are deficient in wastewater treatment and monitoring, the integration of bioprotectant fungi, i.e., *T. viride*, can serve there as an inexpensive, eco-friendly mitigation approach against contaminants and pathogens that harm crop productivity and food security. However, large-scale employment needs strong guidelines, agricultural learning programs, and policy strategies to align agronomic assistance with long-term sustainability and population health considerations.

## 5 Conclusion

The present study reveals that the quality of wastewater strongly determines plant physiological and biochemical responses. Domestic and Lyari wastewater, enriched with essential nutrients, reinforced improved plant growth and yield production. Whereas Malir wastewater, with higher physicochemical stress and heavy metal load, exerted pronounced inhibitory effects on plants. In a contrasting environment, bioprotectant *T. viride* consistently improved plant performance by strengthening physiological functions, decreasing oxidative damage, and upregulating antioxidant activity. The correlation and clustering explorations further exposed coordinated responses between photosynthetic pigments, total soluble proteins, oxidative markers, and defense enzymes, highlighting how *T. viride* regulates multiple pathways rather than a specific attribute. Overall, integration of *T. viride* with quality wastewaters (DWW and LWW) efficiently mitigated both abiotic and biotic stress, inhibited heavy metal absorption, reinforced plant defense mechanisms, and controlled disease, proposing a sustainable approach for a wastewater-derived irrigation system. In practical terms, farmers could integrate *T. viride* by introducing bioprotectant *T. viride* inoculants into irrigation channels and soil amendments through standard mixing or fertigation methods, thereby improving crop resilience while exploiting nutrient-loaded wastewater for irrigation.

Nevertheless, the consequences should be interpreted in light of certain limitations. The study was conducted at a single site and over one growing season. This may restrict the generalizability of the findings. Besides, the study focused on only selected physiological pathways of the crop. The prolonged impacts on soil profile, growth, and yield stability still require evaluation. Therefore, future investigations should include multi-site and multi-seasonal crops to corroborate wastewater suitability across diverse conditions. Assessments of various other physiological pathways and long-term influences on soil nature, microbial dynamics, and crop yield will provide a more comprehensive understanding of wastewater irrigation.

## Data Availability

The original contributions presented in the study are included in the article/supplementary material, further inquiries can be directed to the corresponding author.
